# A Family of CSαβ Defensins and Defensin-Like Peptides from the Migratory Locust, *Locusta migratoria*, and Their Expression Dynamics during Mycosis and Nosemosis

**DOI:** 10.1371/journal.pone.0161585

**Published:** 2016-08-24

**Authors:** Mingyue Lv, Amr Ahmed Mohamed, Liwei Zhang, Pengfei Zhang, Long Zhang

**Affiliations:** 1 Department of Entomology, Key Lab for Biological Control of the Ministry of Agriculture, China Agricultural University, Beijing, P. R. China; 2 Department of Entomology, Faculty of Science, Cairo University, Giza, Egypt; USDA Agricultural Research Service, UNITED STATES

## Abstract

Insect defensins are effector components of the innate defense system. During infection, these peptides may play a role in the control of pathogens by providing protective antimicrobial barriers between epithelial cells and the hemocoel. The cDNAs encoding four defensins of the migratory locust, *Locusta migratoria*, designated LmDEF 1, 3–5, were identified for the first time by transcriptome-targeted analysis. Three of the members of this CSαβ defensin family, LmDEF 1, 3, and 5, were detected in locust tissues. The pro regions of their sequences have little-shared identities with other insect defensins, though the predicted mature peptides align well with other insect defensins. Phylogenetic analysis indicates a completely novel position of both LmDEF 1 and 3, compared to defensins from hymenopterans. The expression patterns of the genes encoding LmDEFs in the fat body and salivary glands were studied in response to immune-challenge by the microsporidian pathogen *Nosema locustae* and the fungus *Metarhizium anisopliae* after feeding or topical application, respectively. Focusing on *Nosema*-induced immunity, qRT-PCR was employed to quantify the transcript levels of *LmDEFs*. A higher transcript abundance of *LmDEF5* was distributed more or less uniformly throughout the fat body along time. A very low baseline transcription of both *LmDEFs* 1 and 3 in naïve insects was indicated, and that transcription increases with time or is latent in the fat body or salivary glands of infected nymphs. In the salivary glands, expression of *LmDEF3* was 20-40-times higher than in the fat body post-microbial infection. A very low expression of *LmDEF3* could be detected in the fat body, but eventually increased with time up to a maximum at day 15. Delayed induction of transcription of these peptides in the fat body and salivary glands 5–15 days post-activation and the differential expression patterns suggest that the fat body/salivary glands of this species are active in the immune response against pathogens. The ability of *N*. *locustae* to induce salivary glands as well as fat body expression of defensins raises the possibility that these AMPs might play a key role in the development and/or tolerance of parasitic infections.

## Introduction

Multicellular organisms continually defend themselves against infection through diverse, effective systems of immunity. Unlike vertebrates, which possess both innate and adaptive arms of the immune system, insects rely on a highly-evolved system of innate immunity [1, 2]. When the latter is activated, it produces a broad spectrum of humoral effector molecules, endowed with a repertoire of defensive mechanisms, including the production of antimicrobial peptides (AMPs) [3]. Insect AMPs can be categorized into three main classes: defensins, cecropins and proline-rich/glycine-rich AMPs [4]. In various sequenced insect genomes, AMP-encoding genes are present as multigene families and are overrepresented in the genomes of some insect species [5]. Defensins are members of a large family of naturally occurring AMPs that contribute significantly to host defense against microbial invasion in animals, including insects and humans, and are also widely produced in plants [6, 7].

Insect defensins are a diverse family of cysteine-rich, small (ca. 5 kDa) cationic, immune-inducible peptides that lack enzymatic activity and possess amphiphilic properties. They are synthesized as precursor propeptides and their sequences mostly form β-pleated sheet structures that are stabilized by three intramolecular disulfide bonds between six conserved cysteine residues linked in the following pattern: C1-C4, C2-C5, and C3-C6 [6, 8]. These disulfide bonds contribute to their antimicrobial and chemotactic activities [9]. According to their structural topologies and sequence homologies, insect defensins can be classified into three main classes (i) defensins with an α-helical domain and two anti-parallel β-strands (αββ scaffold), with the α-helix stabilized by two disulfide bridges to one strand of the β sheet, together, such elements give the structure termed cysteine-stabilized α-helix/β-sheet motif (CSαβ motif), or those with an additional short N-terminal β-strand (βαββ scaffold) that are stabilized by three or four disulfide bridges; (ii) defensin with a triple-stranded antiparallel β-sheet (βββ); and (iii) defensins with a hairpin-like β-sheet structure [4]. Genes of insect CSαβ defensins represent a multigene family in which the copy number varies among insect species [10]. Because of the potential of sequence divergence to rapidly erode signals of homology, particularly within a short sequence, the identification of novel AMPs in a newly sequenced genome is challenging [5]. Hence, computational and functional approaches are required to characterize AMPs across many insect taxa in addition to traditional homology-based methods.

Defensins are mostly active against Gram-positive bacteria; though, broad activity against Gram-negative bacteria, filamentous fungi, and protozoa has been reported previously [11, 12, 13, 14]. Thus, they play multifaceted roles in insect immunity. Interestingly, peptides spanning the carboxy-terminal segment of insect defensins, i.e. without the disulfide bridges and the N-terminal helix region, exhibit varying antibacterial potencies and spectra [15]. It is widely believed that defensins function via disintegrating the bacterial membrane or interfering with membrane assembly, or exert their activity by disrupting the permeability barrier of the outer and inner membranes of Gram-negative bacteria [4, 16, 17]. They are widely distributed across phylogenetically higher insect orders (Diptera, Coleoptera, Hymenoptera, and Lepidoptera) [10]. Defensins have also been identified in phylogenetically lower insect orders like Odonata [18], but have not been characterized from orthopterans, locusts and grasshoppers. Primarily, they have been isolated and characterized from hemolymph, fat body or midgut of immune-induced insects, and scarcely reported from the salivary glands [4, 19].

The migratory locust, *Locusta migratoria*, is a notorious migrating Old World locust of global concern that causes unpredictable outbreaks and a wide host-range of agronomic hosts [20]. New control strategies such as the use of biopesticides based on spores of fungi and microsporidia have been developed [21, 22]. The entomopathogenic fungus *Metarhizium anisopliae* var. *acridum* Driver and Milner (Deuteromycotina: Hyphomycetes) was developed by the LUBILOSA program (Lutte Biologique contre les Locusts et Sauteriaux) to control locusts and grasshoppers [22]. This fungus produces conidia both inside and outside of acridid bodies. When these conidia come into contact with the cuticle of insects, they germinate and penetrate into the hemocoel where they multiply [23, 24]. The glucan recognition proteins (βGRP/GNBP) and persephone (PSH) immunologically recognize fungal molecular patterns (mostly β-1,3-glucans) and virulent factors (PR1) from the growing hyphae. In turn, the insect immune system initiates a series of defenses, such as melanization and encapsulation, including the production of reactive oxygen and nitrogen intermediates, secretion of β-1,3-glucanases, and biosynthesis of AMPs [25, 26, 27].

The unicellular and eukaryotic obligate intracellular spore-forming microsporidian *Nosema locustae* Canning, 1953 (Microsporidia: Nosematidae) is a global orthopteran pathogen with a remarkably wide host range of around 102 species of Orthoptera [21, 28, 29]. It is the only microsporidian that has been developed as a microbial control agent of grasshoppers that is suited for long-term management applications and is transferable from deceased infected grasshoppers [30]. The sporont gives rise to a single small resistant spore, which spontaneously germinates [31]. A mature spore or infective phase is the only stage that occurs outside of the host, and the spore can survive dormant for a long time. The peroral infection of grasshoppers affects the digestive system with infection initiated in the midgut where the spores pierce the midgut wall, and the parasites then spread within the host (nosemosis) [30, 32]. Fat body cells are the predominant site of parasite development, though infection of tracheal epithelium cells and hemocytes also occurs. All stages of the parasite are in direct contact with the host cell cytoplasm (i.e., no interfacial envelopes are present) [31]. *N*. *locustae* pathogenesis results in lethargic locusts with impaired development, mobility, and reproductive performance [33]. Aged grasshopper nymphs are less susceptible to *N*. *locustae* infections than young nymphs [34].

For both *N*. *locustae* and *M*. *anisopliae*, the moderate speed of their insecticidal action is perceived as a potential downside. Nevertheless, combinations of microsporidian and fungal pathogens might enhance the overall efficacy of control processes. Therefore, studies on mechanisms of pathogenesis and host defenses may ultimately contribute to the development of more efficient anti-locust biopesticides. Moreover, the isolation and characterization of a wide range of AMPs are crucial for the continued development of effective control strategies. Recent research highlighted the roles of several immune-related proteins in locusts and how the latter recognize and fight pathogens [27, 35, 36, 37]. No members of the defensin family have so far been characterized from this important insect pest. Additionally, the role of defensins against *N*. *locustae*, or against microsporidia in general, has never been investigated at the molecular basis. Therefore, the main objective of this study was to unravel the role of locust defensins and the mechanisms upregulating their transcriptional expression during mycosis and nosemosis.

## Materials and Methods

### Ethics statement

No specific permits were required for the field collection of the locust, *L*. *migratoria*. The collection locations either in China or Egypt are not privately-owned or protected in any way. This species is widely distributed. It occurs throughout Africa, Asia, Europe, Australia and New Zealand (*cf*. FAO website: http://www.fao.org/ag/locusts-CCA/common/ecg/1078/en/LMI-_Distribution_map3.pdf) and represents a common agricultural pest and is not included in any list of protected animals in China or Egypt.

### Insect rearing and tissue preparation

*L*. *migratoria* were reared on fresh wheat seedlings at 28–30°C, 60% relative humidity with an 18L/6D photoperiod. Fourth instar locusts at 2–3 days after ecdysis were used in all experiments, unless otherwise indicated. Different tissues (fat body, salivary glands, and labial and maxillary palps) were dissected, immediately frozen in liquid N_2_ and stored at -70°C until RNA isolation. All total RNA extractions were performed with TRIzol® reagent (Invitrogen, Carlsbad, CA, USA) according to the manufacturer’s protocols. Total RNA was dissolved in RNase-free water and the RNA quality was measured for yield and purity using a Nanodrop ND-2000 spectrophotometer (NanoDrop products, Wilmington, DE, USA). RNA integrity was checked on an Agilent 2100 BioAnalyzer (Agilent Technologies, Englewood, CO, USA).

### Bacteria, immune challenge, and antibacterial activity testing

*Serratia marcescens* (EMCC 1247) was grown in LB medium (10 g/liter tryptone, 5 g/liter yeast extract, 5 g/liter NaCl) at 37°C with good aeration in a shaking incubator. One hundred microliters of an overnight culture were inoculated into 5 ml of fresh LB medium and the culture was kept shaking until OD_540_ reached 0.8, at which point it was used in an initial experimental challenge assay. Fifth instars *L*. *migratoria* were injected, individually, with 10 μl a phosphate-buffered saline (PBS; Loba Chemie, India) washed *S*. *marcescens* solution (2×10^6^ bacteria/ml). Fat body tissues, 2–4 h post-infection, from three experimental groups, each of 15 nymphs, were prepared according to Mohamed *et al*. [38]. Antibacterial activity of fat body extracts of naïve, PBS-injected, and *S*. *marcescens*-induced nymphs were assessed with a modified version of the plate-growth-inhibition assay of Hultmark *et al*. [39] using both the G^¯^ *E*. *coli* (ATCC 11775) and the G^+^ *S*. *aureus* (ATCC 12600).

### Fungal cultures, inoculation techniques, and tissues preparation

Microsporidia are most closely aligned with Fungi and comprise a separate division of the kingdom Fungi in recent systematics [40, 41], so we used the term "fungal cultures” collectively. The pathogens used in this study were originally established by the Key Laboratory for Biological Control of Pests of the Ministry of Agriculture, College of Biological Sciences, China Agricultural University, Beijing 100193, PR China.

*Metarhizium anisopliae* var *acridum* was cultured on half-strength Sabouraud’s Dextrose agar at 27°C for 10 days under constant light. Conidia were harvested by standard methods and the resulting spore suspension was used. The concentration of spores within the various preparations was determined with a Neubauer hemocytometer. The nymphs were topically inoculated with conidia, the natural route of infection, with a suspension solution of 10^8^ spores in water with 5% Tween 80 (Sigma-Aldrich Corp., St. Louis, MO, USA). Spore suspensions were placed onto the abdominal cuticle of each nymph. The experiments were replicated three times during a 10-days-period.

For inoculations with *N*. *locustae*, water suspensions of spores were added between two pieces of corn leafs as a sandwich for individual locust feeding. Fourth instars were starved for 24 h before treatments were applied. Three groups of locust nymphs, each containing 60 individuals, were used. Insects were individually reared and provided with spores (10^5^ spores/insect). *Nosema* infection in nymphs was examined with a microscope by removing tissues, e.g. alimentary canal or Malpighian tubules, and examining them in wet mount preparations for the presence of spores. The experiments were replicated three times during a three-week-period.

The target tissues, salivary glands and fat body, were dissected and RNA was extracted from 3 nymphs with three replicates for each tissue (3 biological and technical replications) on the 1^st^, 3^rd^, 5^th^, 10^th^ and 15^th^ day after inoculation of *Nosema*, or on the 1^st^, 3^rd^, 5^th^, 7^th^ and 10^th^ day after inoculation of *Metarhizium*. Spatiotemporal expression patterns were examined for each target gene (*LmDEF1*, *LmDEF3*, and *LmDEF5*) in the salivary glands or fat body with non-quantitative RT-PCR. Uninfected insects were used as a negative control, while, the *actin* of *Nosema* and *Metarhizium* were used as positive controls with PCR. For qRT-PCR, experiments were performed with samples collected at the 3^rd^, 5^th^, 10^th^ and 15^th^ day post-*Nosema* inoculation.

### Transcriptome sequencing and analysis

Transcriptome sequencing was employed by standard protocols and then analyzed through bioinformatics. For RNA extraction, maxillary and labial palps were pooled from 30 individuals of the naïve 5^th^ instar (3–5 days old, both male and female) were collected and then total RNA was extracted. The RNA sample was purified and tested for purity and integrity on an Agilent 2100 Bioanalyzer based on electropherogram and RNA Integrity Number (RIN) and finally was introduced into an Illumina HiSeqTM 2500 platform by Biomarker sequencing company (Beijing, China) for standard sequencing. Briefly, mRNA enriched via the NEBNext® Poly(A) mRNA Magnetic Isolation Module (NEB #E7490) were used for library construction (NEBNext® mRNA Library Prep Master Mix Set for Illumina®, E6110 and NEBNext® Multiplex Oligos for Illumina®, NEB #E7500 (Index Primers Set 2)) followed by library quality check on agarose gel and qPCR (Library Quantification Kit for NGS, KK4824, Kapa Biosystems, Inc., MA, USA). Raw paired-end sequencing reads were evaluated and trimmed with a customized Perl script (Biomarker, China) to guarantee high reliability of future assembly and annotation. The remaining high-quality reads were assembled with Trinity v.2.0 software (default settings were applied, except for Min-contig-length (200 bp) and Group-pairs-distance (500 bp)) into contigs according to reads overlapping and then contigs were clustered into transcripts and unigenes using information from paired-end reads and similarity between contigs. Unigenes were annotated and aligned to protein databases from NCBI-nr (non-redundant protein databases), Swiss-Prot, GO (Gene Ontology Consortium), COG (Cluster of Orthologous Groups), and KEGG (Kyoto Encyclopedia of Genes and Genomes). Target protein functional information was compared and assigned from comparative annotation to the most similar protein in those databases. Next, defensin genes within the dataset were identified.

### Identification of *L*. *migratoria* defensin sequences (LmDEFs) in the palpi transcriptome

Two approaches were employed: (i) We searched the publicly available NCBI *L*. *migratoria* EST database (LocustDB) derived from various body parts [42] for any defensin precursors using various defensin sequences (marked in bold in S1 Table); (ii) We used a collection of insect defensin sequences (S1 Table) to generate a BLAST database in NCBI Genome Workbench 2.8.10 (available at http://www.ncbi.nlm.nih.gov/tools/gbench/), and carried out tblastx queries with a cut-off of 10^−5^ against our database using *L*. *migratoria* palpi transcriptome data, or our library of the already assembled and annotated unigenes. Identified hits indicating candidate defensin sequences (= all single target unigenes; obtained after all the repeatedly aligned defensins unigenes were removed until only one remained) were used to re-tblastx the NCBI nr database to verify identity. Several sequences annotated as insect defensin or defensin-like peptide were identified, which were used as queries to perform tblastx again with the *L*. *migratoria* palpi transcriptome database. Finally, both ends of each unigene ORF structure were predicted.

### Expression dynamics

#### Expression of *LmDEFs* in different tissues in a time-series

Quant cDNA Synthesis Kit (Tiangen, Beijing, China) was used for reverse transcription of total RNA with 1 μg total RNA as template. Non-quantitative RT-PCRs were performed with gene-specific primers. All the predicted defensins were verified by the conventional RT-PCR with the primers listed in S2 Table (Lmig-ORF 1, 3–5). The integrity of the cDNA preparation was tested with primers for the *L*. *migratoria* actin gene [Genebank: AY344445.1]. PCR products were run on 1.2% agarose gels and visualized by ethidium bromide staining. The PCR program began with an initial denaturation at 95°C for 5 min. This was proceeded by 10 cycles of denaturation at 94°C for 30 s, annealing at 68–59°C (-1°C/c) for 30 s, and extension at 72°C for 1 min, which was followed by 20 cycles of denaturation at 94°C for 30 s, annealing at 58°C for 30 s, and extension at 72°C for 1 min. Then, there was a final extension at 72°C for 5 min.

#### Relative quantification of gene expression post-infection with *Nosema* by reverse transcription-real time-PCR (qRT-PCR)

Total RNAs extracted from the fat body and salivary gland frozen tissues were reverse transcribed with the Quant cDNA Synthesis Kit (Tiangen, Beijing, China) according to the manufacturer’s protocol. PCR amplifications were carried out on a 7900HT Fast Real-Time PCR System (Applied Biosystems, Carlsbad, CA, USA) with KAPA SYBR® FAST qPCR kit (Kapa Biosystems, Wilmington, MA, USA). Briefly, the following components were mixed to obtain a final volume of 20 μl: 1 μl reverse-transcribed RNA, 10 μl of SYBR® FAST qPCR master mix (2x) containing 5 mM MgCl_2_, 0.4 μl ROX high (50x), 0.3 μl of each primer (5 pmol each) and up to 20 μl RNAse-free water. The following PCR amplification program was used for each pair of primers with various cDNAs as the templates consisted of the following steps: (i) initial denaturation at 95°C for 3 min; (ii) an amplification and quantification program consisting of 40 cycles (95°C for 15 s, 60°C for 20 s, and 72°C for 15 s with a single fluorescence measurement and a temperature transition rate of 20°C/s); (iii) a melting curve program (72 to 95°C with a heating rate of 0.1°C/s and a continuous fluorescence measurement), and (iv) final a cooling step to 30°C. Negative controls consisted of no cDNA template (water) reaction mixtures and were run with every assay. The specificity of the reaction was checked by analyzing the melting curve of the final amplified product. Primers were designed based on the complete ORF of the target genes using Primer-3 software (S2 Table) and amplification efficiencies determined (S4 Fig). Specific gene transcript levels were normalized to the housekeeping gene *Lmβ-actin* and expressed as a function of the reference condition (naïve nymphs) according to the 2^−ΔΔCt^ [ΔΔCt = ΔCt(test sample)–ΔCt(calibrator)] method [43]. Three PCR replicates were run on each experimental condition with 3 nymphs/pool per treatment per replicate. The final values reported are the averages of three independent experiments. PCR products ran on agarose gels were operated to confirm the formation of a single product of the desired size. For a single gene, data sets were analyzed to determine whether they were normally distributed. To test the differences within gene transcript levels across time, a one-way ANOVA with Tukey's post-hoc analysis was used. The non-parametric Kruskal-Wallis test was applied when data did not fit a normal distribution. In all cases *p*<0.05 were considered significant. All statistical analyses were performed using SPSS 22.0 software (IBM SPSS Statistics for Windows—IBM Corp., Armonk, NY, USA).

### Bioinformatics, phylogenetic analysis, and protein 3D structure prediction

Multiple sequence alignments were performed with ClustalW algorithm of Kalign at EMBL-EBI server and edited manually from their amino acid alignment. The phylogenetic analysis was performed with multiple aligned sequences with a neighbor-joining distance method in MEGA6 for Windows [44]. Theoretical isoelectric points were calculated by the Compute pI/Mw tool of ExPASY (http://au.expasy.org/). Signal peptide and motif/domain prediction and annotation were determined with Smart software [45].

LmDEFs homology modeling was performed with four different protein modeling servers (Swiss-Model: http://swissmodel.expasy.org/, Phyre2: http://www.sbg.bio.ic.ac.uk/phyre2/, Raptor X: http://raptorx.uchicago.edu/, and I-Tasser http://zhanglab.ccmb.med.umich.edu/I-TASSER/). The models were validated using Structural Analysis and Verification Server (SAVES: http://services.mbi.ucla.edu/SAVES/). The models presented in this study, based on the SAVES validation, were the Raptor X predicted ones. The comparative three-dimensional structure models were optimized by PyMOL software package (http://www.pymol.org). The PDB entries # 1icaA, 1ica, 1aodA, and 4uj0A were used as the templates for LmDEF 1, 3–5, with corresponding p-values of 2.24e-04, 1.85e-04, 1.11e-02, and 8.36e-03, respectively. Afterward, PDB files of the modeled LmDEFs 3D structure were submitted to the ProFunc server (http://www.ebi.ac.uk/thornton-srv/databases/profunc/) [46] to identify the likely biochemical functions. The server used a series of methods, including fold matching, residue conservation, surface cleft analysis, and functional 3D templates to identify the possible protein function. The electrostatic potential was calculated using the Poisson-Boltzmann continuum electrostatics equation implemented in PyMOL.

## Results

### Immune challenge

Growth inhibition assays were performed to detect the presence of antimicrobial activities in the fat body of 5^th^ instars at 4-h post-immune challenge with *S*. *marcescens*. Both *E*. *coli* and *M*. *luteus* were susceptible and antibacterial activities against both bacteria were observed pointedly in tissues from induced nymphs, and to a lesser extent from unchallenged or PBS-injected nymphs (S1A Fig). Fat body preparations from induced instars were checked by SDS-PAGE (S1B Fig). Two bands with molecular masses of around 11 and 5 KDa, which were below the size of the *L*. *migratoria* 15.7 kDa c-type lysozyme (GenBank acc. AHE76131.1), were observed. The results indicated the presence of molecules that exhibited molecular masses closer to that of AMPs such as defensins.

Mining of LocustDB and subsequent tblastx searches resulted in the identification of a partial defensin cDNA fragment (CO837175.1). No obvious defensin orthologs were found for *ca*. 40% of the *L*. *migratoria* EST sequences. This may be explained by the fact that EST data do not essentially cover the entire protein-encoding region of a gene. TBLASTn search with this fragment as query showed 43 and 42% identities (E_value_ 1.5 and 0.96) to the defensin precursors of both *Pediculus humanus corporis* (GeneBank: XP_002428138.1) and *Graminella nigrifrons* (GeneBank: AIY24634.1), respectively. Induction of defensin transcripts in fat body cells by *Serratia* was also confirmed by non-quantitative RT-PCR based on this partially identified fragment (1^st^ primer pair in S2 Table). Defensin transcript levels were increased 2 hr after infection. Furthermore, upregulation of defensin expression by infection was also observed (S1C Fig).

### Identification of defensins in locust by transcriptome-targeted analysis

**Fig 1:**
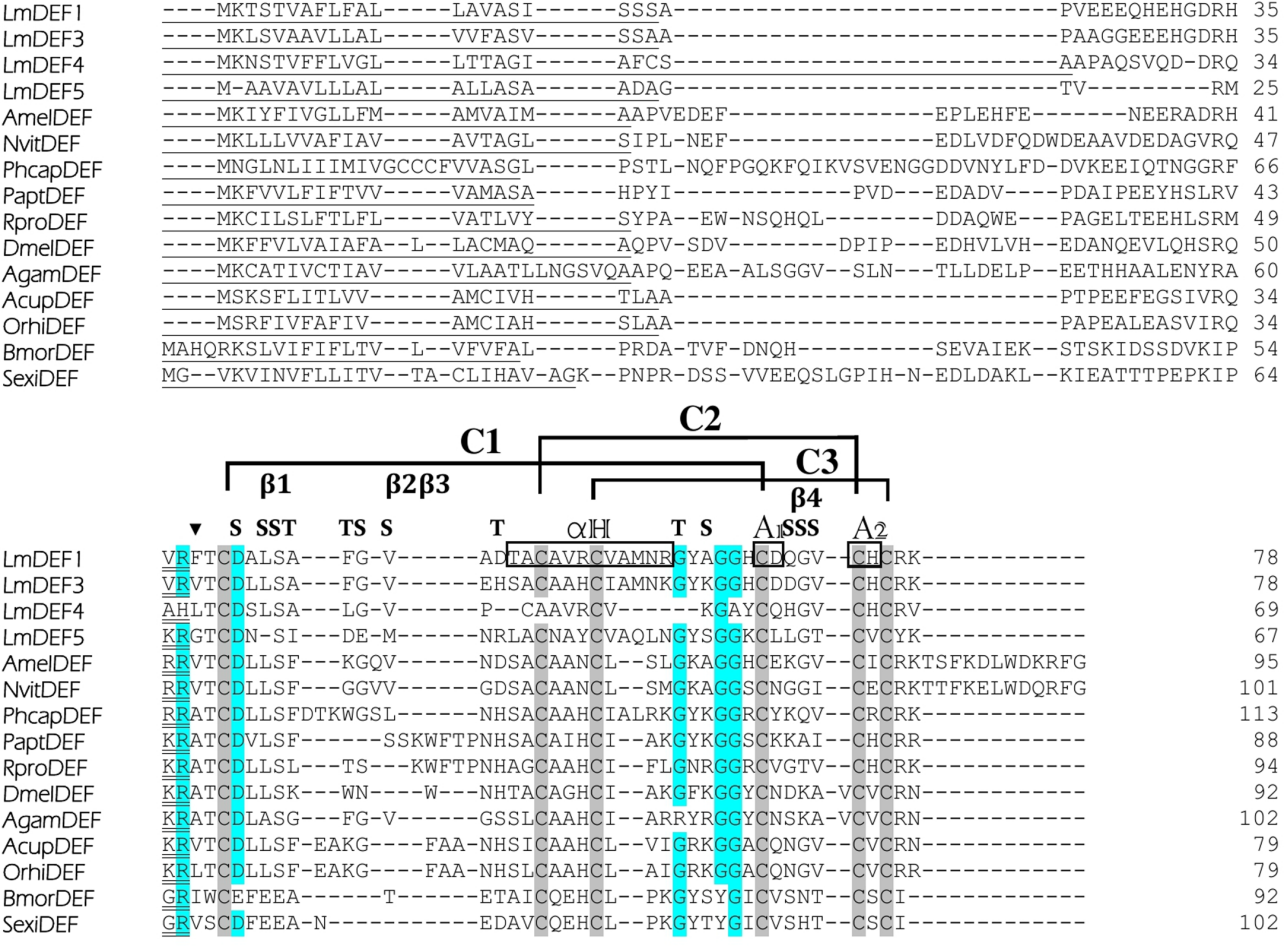
Multiple alignments of newly identified LmDEFs with other insect CSαβ defensins. Residues conserved in >50% of proteins are shaded. The numbers to the right refer to the position of the last residue of each line. Signal peptides are underlined. Bars indicate gaps to optimize the alignments. The six conserved cysteine residues involved in disulfide bridges are gray shaded. Possible activation peptide cleavage sites are marked with a triangle; enzymatic processing sites (e.g. -KR↓) to release the mature peptides are double underlined. The latter was predicted and/or determined based on various bioinformatical tools (PeptideCutter prediction, the cleaver package, and others) and similarity searches with mature insect defensins from GenBank and reported in the literature. The secondary elements (loop, α-helix, and β-sheet) of insect defensins are indicated as follow: β1, β2, β3, and β4 are regions for potential β turns; A1 and A2 are regions for potential β strands; H is a region for potential α-helix; C1, C2, and C3 are the potential disulfide linkages; S are the regions for bends; T are regions for hydrogen-bonded turns of CSαβ defensins (Following Dassanayake *et al*. [10]). Accession numbers for the selected insect defensins: AmelDEF, *Apis mellifera*: [GeneBank: NP_001011616.2]; NvitDEF, *Nasonia vitripennis*: [GeneBank: NP_001159944.1]; PhcapDEF, *Pediculus humanus corporis*: [GeneBank: XP_002432619.1]; PaptDEF, *Pyrrhocoris apterus*: [GeneBank: AGI17576.1]; RproDEF, *Rhodnius prolixus* [GeneBank: AAO74626.1]; DmelDEF, *Drosophila melanogaster* [GeneBank: AAO72500.1]; AgamDEF, *Anopheles gambiae* [GeneBank: ABB00983.1]; AcupDEF, *Anomala cuprea* [GeneBank: BAD77967.1]; OrhiDEF, *Oryctes rhinoceros* [GeneBank: BAA36401.1]; BmorDEF, *Bombyx mori* [GeneBank: BAG48202.1]; SexiDEF, *Spodoptera exigua* [GeneBank: AEW24427.1].

Transcriptome sequencing of labial and maxillary palpi yielded 71,994,198 paired reads with total nucleotide bases number of 14.54 Gb. High Q30 value of 82.28% and moderate GC content of 49.15% indicated a high sequencing quality. Analysis of transcriptome data identified four homologous unigenes encoding putative defensins within the insect defensin family (IPR017982). All of them were predicted to be full length, with full ORF sequence ranging from 204–237 bp, and all of them had a signal peptide (S2 Fig). The complete coding sequences were deposited in GenBank and designated as *LmDEF1* [Genebank: KU516092.1], *LmDEF3* [Genebank: KU516094.1], *LmDEF4* [Genebank: KU516095.1], and *LmDEF5* [Genebank: KU516096.1] (Note—*LmDEF2* was initially submitted to GenBank but was subsequently shown to be an unverifiable sequence). The lengths of the full protein sequences were 78, 78, 69, and 67 amino acid residues, respectively (Fig 1). The theoretical isoelectric points (pI)/molecular weights (Mw; kDa) were 6.48/8.29, 6.56/8.37, 8.23/7.19, and 8.27/6.93, respectively. Sequence identities/similarities among the different structural forms of LmDEFs are presented in Table 1.

**Table 1:** Identity and similarity values among the four putative locust defensins.

Form	LmDEFs	Identity				LmDEFs	Similarity			
		LmDEF1	LmDEF3	LmDEF4	LmDEF5		LmDEF1	LmDEF3	LmDEF4	LmDEF5
**Precursor**	LmDEF1	**---**	65.4	48.1	37.3	LmDEF1	**---**	74.4	54.4	49.3
	LmDEF3	**65.4**	**---**	42.5	38.7	LmDEF3	**74.4**	**---**	53.8	48.0
	LmDEF4	**48.1**	**42.5**	**---**	30.8	LmDEF4	**54.4**	**53.8**	**---**	36.9
	LmDEF5	**37.3**	**38.7**	**30.8**	**---**	LmDEF5	**49.3**	**48.0**	**36.9**	**---**
**Prodefensin**	LmDEF1	**---**	70.2	46.4	44.4	LmDEF1	**---**	77.2	51.8	48.9
	LmDEF3	**70.2**	**---**	45.6	44.4	LmDEF3	**77.2**	**---**	54.4	51.1
	LmDEF4	**46.4**	**45.6**	**---**	29.7	LmDEF4	**51.8**	**54.4**	**---**	35.1
	LmDEF5	**44.4**	**44.4**	**29.7**	**---**	LmDEF5	**48.9**	**51.1**	**35.1**	**---**
**Mature form**	LmDEF1	**---**	75.0	53.8	45.0	LmDEF1	**---**	82.5	59.0	50.0
	LmDEF3	**75.0**	**---**	51.2	45.0	LmDEF3	**82.5**	**---**	58.5	52.5
	LmDEF4	**53.8**	**51.2**	**---**	29.7	LmDEF4	**59.0**	**58.5**	**---**	35.1
	LmDEF5	**45.0**	**45.0**	**29.7**	**---**	LmDEF5	**50.0**	**52.5**	**35.1**	**---**

Protein sequence identities and similarities were calculated with the EMBOSS Water tool at EMBL-EBI server using the Smith-Waterman algorithm. Values are expressed as %.

In addition to the Pfam search, analysis of the four full peptide sequences with the SMART server (http://smart.emblheidelberg.de/) [45] showed that, except for LmDEF4, they all belong to the arthropod defensin family. All of them contain a Knot1 domain (Table 2) which is characteristic to knottins or cystine-knot peptides, including the antibacterial defensins and the scorpion alpha-neurotoxins, a feature which was reported for some insect defensins, for instance, in *Galleria mellonella* [47] and *Cotesia vestalis* [48].

**Table 2:** Analysis of some predicted domains and motifs of the putative LmDEFs.

Feature	LmDEF1			LmDEF3			LmDEF4			LmDEF5		
	Start	End	E-value	Start	End	E-value	Start	End	E-value	Start	End	E-value
**signal peptide**	1	21	N/A	1	21	N/A	1	23	N/A	1	20	N/A
**Pfam:Defensin2**	45	77	0.0000048	45	77	1.1e-9	*N/A*			35	66	1000
**Knot1 domain**	39	78	0.256	39	78	0.0139	38	68	195	29	67	9.85

### Structural analysis of the peptides

Multiple sequence comparison with the retrieved full-length insect defensin sequence and topology analysis revealed three different regions: signal peptide, prodefensin and the mature defensin (Fig 1). Significant sequence diversity was observed in the signal peptide and prodefensin regions perhaps in part because of ambiguities like amino acid substitution, insertion, and deletion. Based on the sequence homology and alignment to structurally known CSαβ mature defensins with six strictly conserved cysteine residues all involved in intrachain disulfide bonds formation using the Dassanayake *et al*. [10] scheme, LmDEFs can be classified as CSαβ defensins that comprises three apparent regions: an N-terminal loop comprising 3 β-turns and one γ-turn, a central amphipathic α-helix, and a C-terminal antiparallel β-strand consisting of one β-turn and a β hairpin (Fig 1). Sequence comparisons, with few exceptions, revealed relative conservation of glycines, arginine, and aspartate (shaded). These defensins were also observed to have a variable region that is a 4^th^ β-turn, where the amino acid substitutions are seen. The predicted locations of putative domains within the sequences are indicated in Fig 1. Interestingly, LmDEF4 lacks the 2^nd^ well-conserved C2 that forms with C5 the second disulfide bond required for stabilizing the peptide tertiary structure, and also has been reported to be necessary for defensin activity [9]. Proteins lacking this essential amino acid are called defensin-like peptides (DLPs), and have been described in some insects [49, 50, 51]. Defensin-like genes play an important role in both immunity and metamorphosis [51].

**Fig 2:**
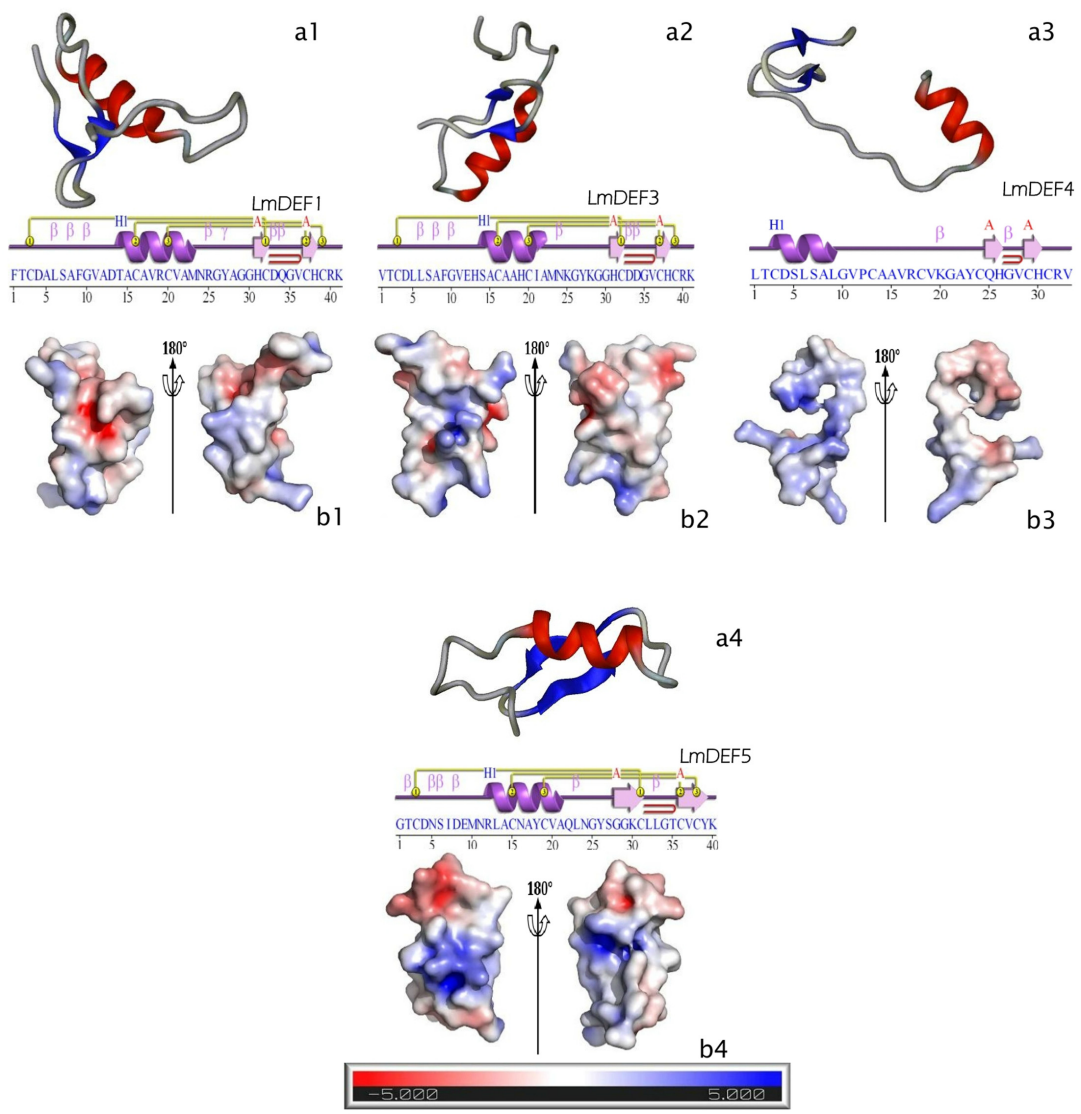
Homology modeling of LmDEFs. a, Representation of the homology-derived solution structure of LmDEFs (a1-a4). α-helix (red), strand or β-sheet (blue), and coil (gray); the images were optimized with the Protein Picture Generator v1.21. Secondary structure elements were predicted with ProFunc server at EMBL-EBI; key: Helix-Strand: purple; helices labelled H1, H2,…and strands by their sheets A, B,…*Motifs*: β beta, turn γ gamma turn, and beta hairpin (red arch). Disulfide bond (1–1, 2–2, 3–3). b, Electrostatic potential distribution on the peptide surfaces (b1-b4). Positive potential is shown in blue, and negative potential is in red; the contouring value of the potential is in kT/e; the bar at the bottom (-5 to +5). The images were drawn using PyMOL Molecular Graphics System (DeLano Scientific, San Carlos, CA).

3D structural modeling and secondary structure predictions confirmed the CSαβ defensin topology for all LmDEFs (Fig 2 and S3 Table), except LmDEF4, which contains no defensin 2 signature (Table 2). The common motifs are referred to in the 3D models (Fig 2A_1-4_, upper panels) and secondary structure elements prediction (Fig 2A_1-4_, lower panels). It comprises one α-helix (red), two antiparallel β-sheets (blue), and variable random coils (in gray) located at both terminal ends and at regions between the α-helices and β-sheets (taking in account the 3D models). Interestingly, LmDEFs were predicted to have a β hairpin motif (Fig 2A_1-4_, lower panels). The presence of this beta-hairpin with two adjacent disulfides (knottin domain) is essential for antimicrobial activity and has been attributed to the membrane disruptive activity of insect defensins [52]. The electrostatic potential maps of LmDEFs shown in Fig 2B_1-4_ reveal that LmDEFs 1 and 3 have more or less acidic surfaces (red), reflecting the presence of the Asp (D) residues compared to the positive Lys (K) residue at the same position in LmDEF5. LmDEFs sequences, based on their PDB files, were subjected to analysis on the ProFunc server (and EBI-SAS server) to identify their likely biochemical function from their modeled three-dimensional structures. The putative biological processes predicted by this tool were defense and/or stress response, whereas the biochemical functions were mainly predicted to be related to ion channel inhibition.

**Fig 3:**
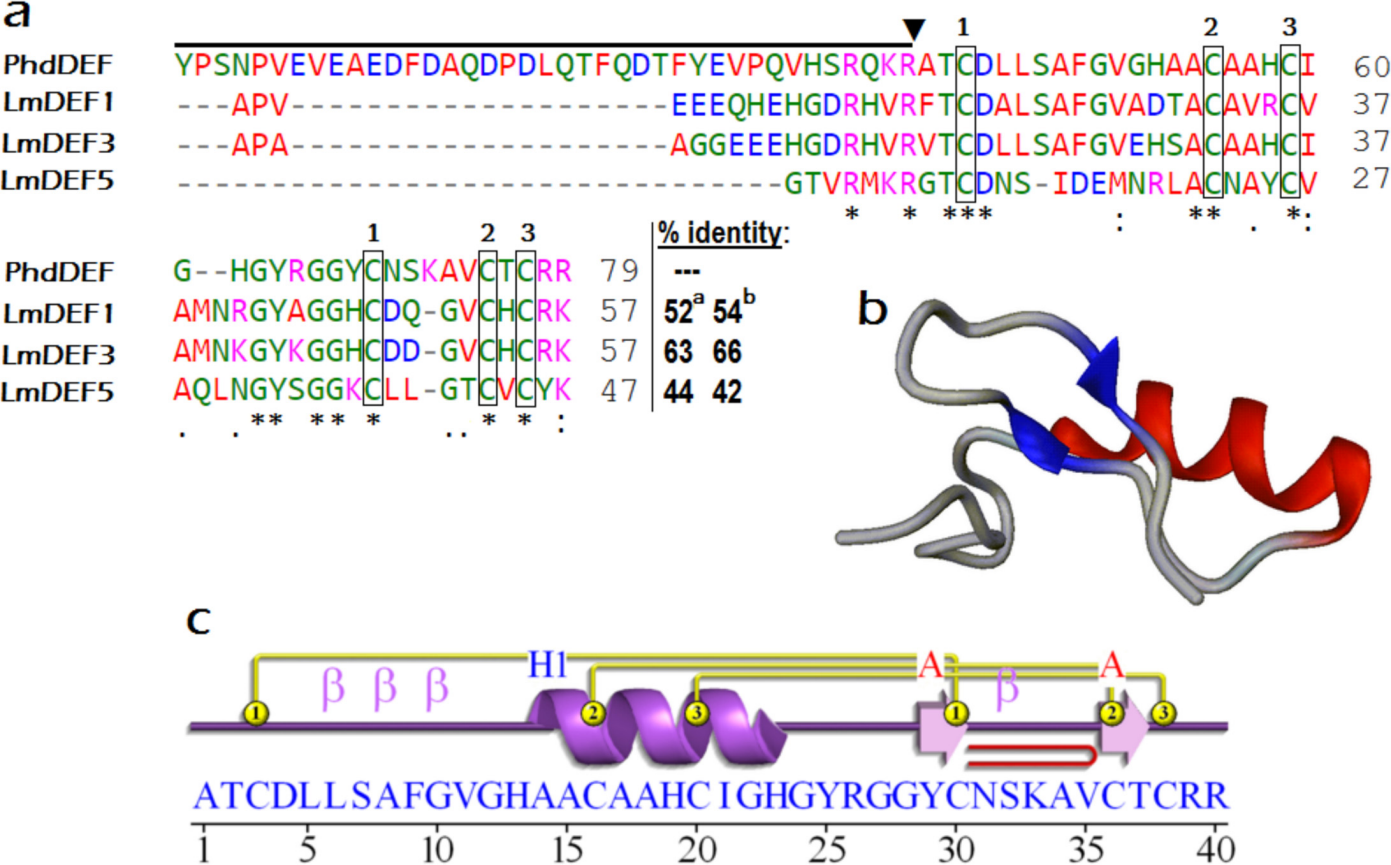
Structural homology of LmDEFs to known antiprotozoal defensin. a, Alignment of LmDEF1, -3, and -5 with the antiprotozoal *Phlebotomus duboscqi* defensin (PhdDEF: Boulanger *et al*. [11]). Percentage identity of LmDEFs to PhdDEF are shown at the end of each individual sequence; ^a^ denote the % identity with PhdDEF full peptide excluding the signal peptide, while ^b^ is denoting that % with the PhDEF mature active peptide. The amino acid residues are colored according to their physicochemical properties (red: small+ hydrophobic [incl. aromatic -Y]; blue: acidic; magenta: basic–H; green: hydroxyl + sulfhydryl + amine + G). The symbols under the alignment indicate: (*) identical sites; (:) conserved sites; (.) less conserved sites. The boxes indicate the six conserved cysteines; the conserved disulfide bridges are shown by # above these boxes (1–1; 2–2; 3–3). The active peptide cleavage site in PhdDEF is marked with a triangle; while the prodefensin is marked by a line. b, Three-dimensional *in silico* structure of “active” PhdDEF based on PDB entry 1icaA (defensin A of *Protophormia terraenovae*) as a template. The homology modeling was carried out with RaptorX; 40(100%) residues were modeled (*p*-value 9.45e-05). c, The secondary structure elements of PhdDEF predicted by ProFunc server for the purpose of comparison between it and LmDEFs (reported in Fig 2). It comprises 1 sheet, 1 beta hairpin, 1 beta bulge, 2 strands, 1 helix, 4 beta turns, and 3 disulfides.

In an attempt to correlate the antiprotozoal activation of LmDEFs biosynthesis (upregulation of *LmDEFs*) with the structure of peptides, a comparison between PhdDEF [11] and LmDEFs 1, 3, and 5 was undertaken (Fig 3). Comparative analysis of the amino acid sequences of PhdDEF and LmDEFs 1, 3, and 5 showed that the latter have shared identities of 54, 66, and 42%, respectively, with PhdDEF. Also, positive charge distribution (attributed mainly to H, K, and R; Fig 3A) in LmDEFs is closely related to PhDEF. Analysis of homology models obtained for the PhdDEF (Fig 3B and 3C) and LmDEFs (Fig 2A_1-4_) in combination with the alignment analysis showed that the LmDEFs shared the common structural features of PhdDEF. Most of the amino acid differences occurred in the α-helical regions of the peptides. These amino acid substitutions did, however, result in a difference of the predicted surface properties between the PhdDEF and the 3 peptides. LmDEFs are more amphipathic in nature, whereas PhdDEF is more polar in the regions surrounding the α-helix.

### Phylogenetic analysis

**Fig 4:**
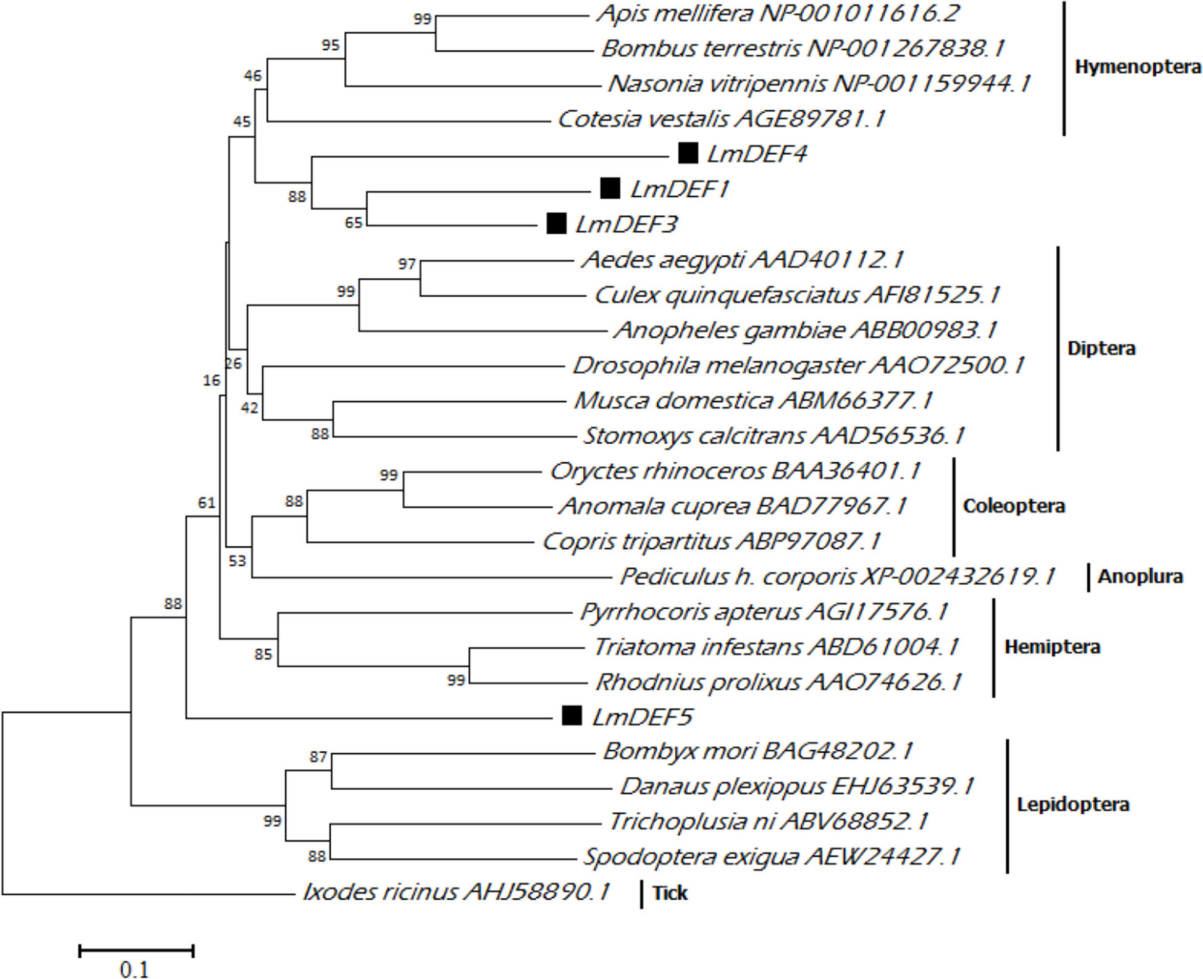
Phylogenetic analysis. The relationship between LmDEFs with other insect defensin was inferred using the Neighbor-Joining method. *Ixodes ricinus* defensin 2 (tick) was used as the out-group. LmDEFs are marked with solid squares. Accession numbers are written next to insect species. Only representatives for defensin-2 were used. More closely related insect defensins within this group were removed to facilitate phylogenetic analysis and representation; therefore, only different lineages of the defensin2 family were shown. Bootstrap (1000 replicates) values are indicated for each root. The evolutionary distances were computed using the p-distance method and are in the units of the number of amino acid differences per site. The analysis involved 26 amino acid sequences that were aligned by Kalign. All ambiguous positions were removed for each sequence pair. The evolutionary analysis was conducted with MEGA6.

Phylogenetic analysis of several more closely-related insect defensins (Fig 4) confidently grouped their sequences into two main lineages, one group comprised of lepidopteran defensins and a second comprised of defensins from Hemiptera, Coleoptera, Diptera, Anoplura, and Hymenoptera. The topology of the phylogram indicated that LmDEFs are diverse, suggesting a variation in their immune mechanisms, phylogenesis, and effector pathways. LmDEF1, LmDEF3, and LmDEF4 are localized within the Hymenoptera cluster, however, LmDEF5 is most closely aligned to the exopterygotes.

### Spatiotemporal expression patterns of *LmDEFs* post-infection with *Nosema* and *Metarhizium*

**Fig 5:**
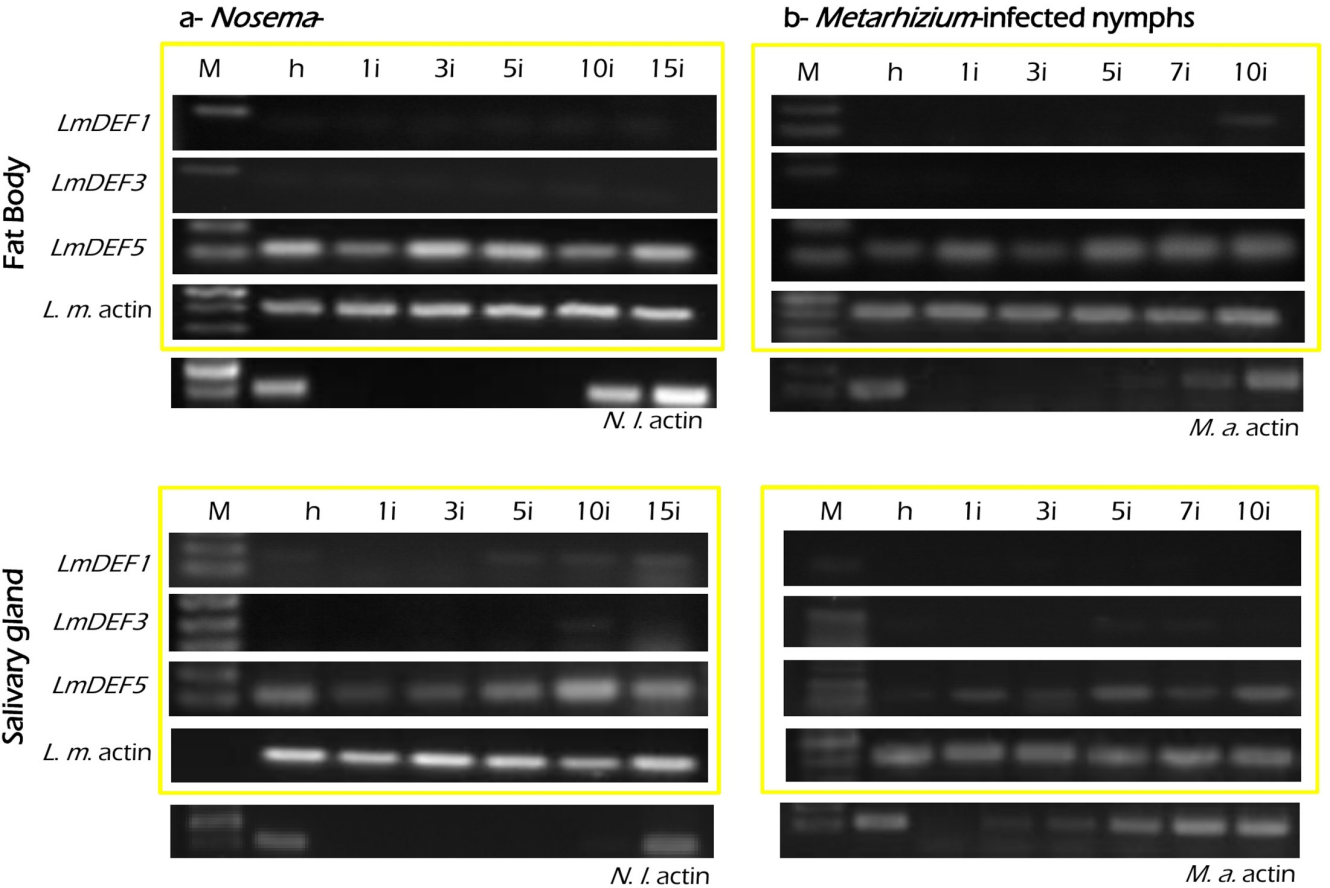
Tissue specificity and developmental expression patterns of *LmDEFs*. a, spatiotemporal expression of *LmDEFs* in *N*. *locustae* infected fat body and salivary gland cells. b, same, but with *M*. *anisopliae*. M, DNA ladder; h, healthy insect; 1i, 3i, 5i,7i, 10i, and 15i the RNA of tissues collected on the 1^st^, 3^rd^, 5^th^, 7^th^, 10^th^, and 15^th^ days after inoculation with pathogens; *L*.*m*. actin, locust actin gene. *N*.*l*., *Nosema* spores and *M*.*a*, *Metarhizium* hyphae as positive controls with RT-PCR. The locust actin gene was used as a control for the integrity of the cDNA templates. Amplification products were analyzed on agarose gels and visualized by UV illumination after ethidium bromide staining. All tissues were dissected from gregarious locusts.

The expression patterns of *LmDEFs* after inoculation with *N*. *locustae* and *M*. *anisopliae* were assessed in the fat body and salivary glands by non-quantitative RT-PCR and compared (Fig 5). *LmDEF4* was not detected upon infection in the fat body and salivary glands; hence, *LmDEF1*, *LmDEF3*, and *LmDEF5* were only tested in the experimental setup. Only *LmDEF5* was uniformly expressed, with slight differences in intensities, in both the fat body and salivary gland cells of both the healthy and pathogens-inoculated 4^th^ instars. In *Nosema*-inoculated nymphs, *LmDEF1* and *LmDEF3* were not detected in the fat body. The transcript level of *LmDEF1* in salivary glands gradually increased from the 1^st^ day to the 15^th^ day after inoculation, whereas *LmDEF3* was detected only in the samples collected at the 1^st^ day and the 10^th^ day after inoculation. In *Metarhizium*-inoculated nymphs, *LmDEF1* was detected in fat body cells from the samples collected at the 10^th^ day after inoculation, whereas *LmDEF3* was not. In salivary gland cells, *LmDEF1* and *LmDEF3* were detected in both healthy and infected insects on the 7^th^ day and on the 5^th^ and 7^th^ days after treatment, respectively.

### Transcriptional profile of defensin gene following *N*. *locustae* infection

**Fig 6:**
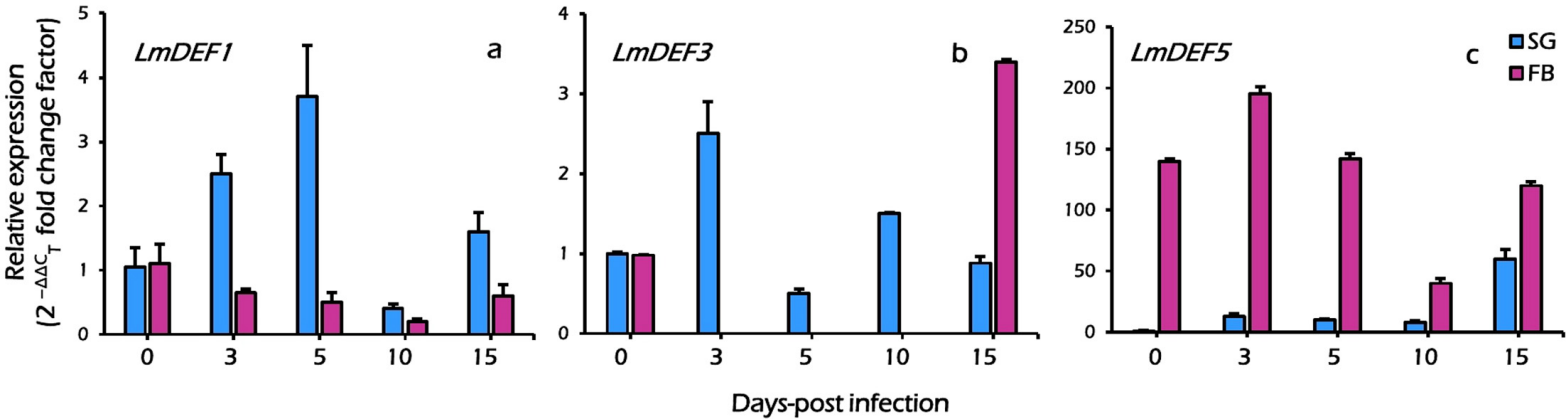
Real-time quantitative PCR profile of *LmDEF* transcripts following *N*. *locustae* infection in the fat body (FB) and salivary glands (SG) in relation to time. Transcript accumulation values were normalized to the constitutively expressed β-actin gene and expressed as a function of the reference condition according to the 2^−ΔΔC^_T_ method. The bars indicate the relative changes in RNA levels compared with the average expression of each gene under non-infected control conditions. The error bars indicate the standard errors of the means. The results are the means of at least three independent experiments. The statistical annotations, including the letters indicating significance, among treatments are given in S4 Fig.

**Fig 7:**
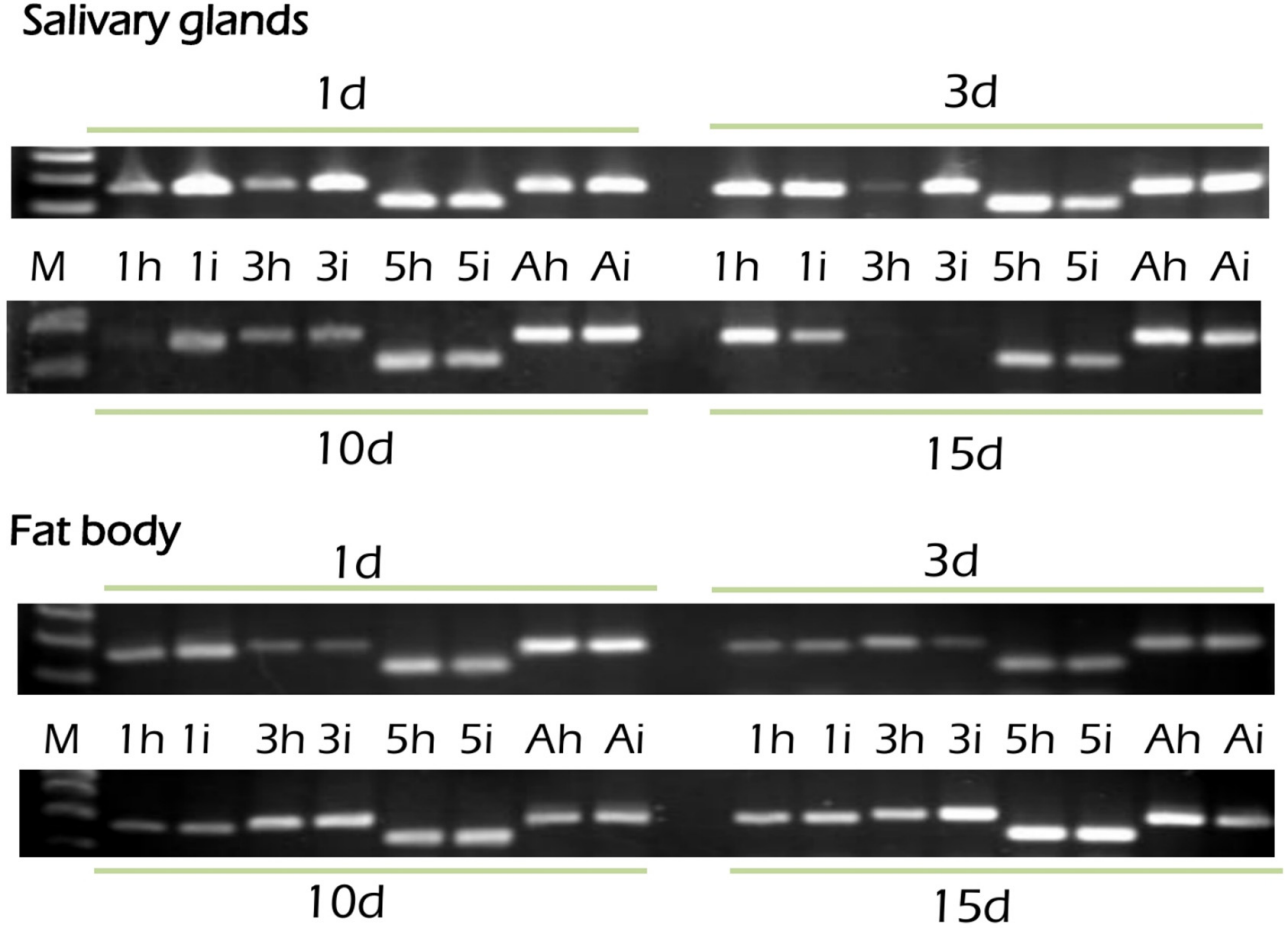
The expression pattern of *LmDEFs* in the fat body and salivary glands in locusts infected with *Nosema* on the 1^st^, 3^rd^, 10^th^, and 15^th^ days post-infection in comparison to control at each time. PCR products, using qRT-PCR primers (S2 Table), were ran on 1.2% agarose gels and visualized by ethidium bromide staining. M, DNA-ladder; numbers preceding characters are gene numbers in healthy locust (h), or infected locust (i); A-actin gene.

Defensins are peptides that are inducible upon microbial challenge. Thus, we examined whether the expression of *LmDEF1*, *LmDEF3*, and *LmDEF5* is subject to pathogen-dependent regulation at the transcriptional level with respect to onset of infection and time series. The expression profile of *LmDEF1*, *LmDEF3*, and *LmDEF5* in *N*. *locustae*-challenged 1- to 15^th^-day-old nymphs (spanning both 4h^th^ and 5^th^ instars) was examined quantitatively, using qRT-PCR analysis, in the fat body and salivary glands (Fig 6). With special reference to the relative quantitative levels of the gene expression patterns in *Nosema*-infected locusts, we also tracked the expression profile of the tested *LmDEFs* in comparison with a naïve control at each time point (Fig 7), using the primers for qRT-PCR that are listed in S2 Table. In our experiments, expression of *Lmβactin* was deemed to be stable, and thus was used for normalization under infection conditions (geNorm) as also supported by its reported E and R^2^ of 2.15 and 1.0, respectively (S3C Fig).

*LmDEFs* were differentially expressed depending on the tissues and time. As shown in Fig 6A and S4 Fig, the transcript levels of *LmDEF1* is nearly the same in both fat body and salivary glands in control specimens. In response to nosemosis and developmental time, *LmDEF1* appeared to gradually increase and was radically elevated at the 5^th^ day post-infection in salivary glands (3-fold change), and then decreased to the levels of control insects. Nevertheless, the transcript levels in fat body decreased with time progression, a change which may be either transient or stable due to the utilization of the fat body of the hoppers by developing spores. The results showed that *LmDEF3* was not induced in fat body at 1–10 days post-infection (p.i.) and its transcripts were nearly absent compared to their prominent levels detected in the salivary glands. However, transcript levels were detected with a moderately higher level at day 15 post infection. In salivary glands, the transcript level of the gene peaked on the 3^rd^ day post-infection, then decreased with time (Fig 6B and S4 Fig). *LmDEF5* was expressed at a comparatively much higher level in the fat body than in the salivary glands. In fat body, the transcript level reached its peak at the 3^rd^ day after inoculation, then decreased. In salivary glands, the expression showed a gradual increase and then peaked (up to more than 40-fold) at day 15 post-treatment. Interestingly, with this tissue, with timespan post-infection, *LmDEF5* transcripts remained at approximately basal levels up to 10 days-post-infection, indicating that the slight downregulation observed with infection was a result of a transient response. However, on the 15^th^ day-post infection it was strongly upregulated (more than 40-fold) (Fig 6C and S4 Fig).

The results revealed that the tested *LmDEFs* are inducibly-expressed, although at rather low levels for *LmDEF1* and *LmDEF3*. With the exception of *LmDEF5*, the results suggest that transcription of *LmDEFs* is upregulated when salivary glands are exposed to an extended period of infection. *LmDEF5* was expressed at markedly elevated levels compared to the other genes (see S4 Fig). A delayed gene overexpression profile with time was detected for *LmDEFs* 3 and 5 suggesting the presence of stress recovery which could be linked to the lower mortality rates (data not shown) of morbid locusts. Hence, *LmDEFs* may act cooperatively to tolerate *Nosema* infection and may have a synergistic effect on resisting the microsporidia in a tempo-spatial expression pattern.

Taken together, the results revealed that these three *LmDEFs* may specifically respond to immune-challenge by *Nosema* with time in the fat body and salivary glands. All three genes are transcriptionally regulated. *LmDEF5* is mainly in the fat body at high transcript levels and gradually increased in salivary glands and to a lesser extent in the fat boy where it then decreased. Whereas *LmDEF* 1 and 3 are expressed in the fat body and salivary glands, and behaved differently in these two tissues. These indicate that the dynamics of three *LmDEFs* after inoculation with *Nosema* may play different roles in relation to phases of *Nosema* in the locust body.

## Discussion

Defensins are crucial components of the immune response of insects against parasitic and pathogenic infections [53]. For instance, it was observed that impairment of honeybee immunity in response to the microsporidian *N*. *ceranae* infection was related to a significant downregulation of several immune genes, including defensins, which favor parasite development [54, 55]. Likewise, survival assays of DEF1-silenced mosquitoes injected with *S*. *aureus* bacteria showed that DEF1-silenced mosquitoes were highly susceptible to *S*. *aureus* (20% survival) at 7 days post-challenge [56]. Defensins are expressed in fat body cells, hemocytes, midgut epithelium, and epidermal cells [4].

### Defensins and their roles in locust

In the present study, a group of defensins was identified and characterized from the transcriptome of *L*. *migratoria*. At the structural level, the full-length peptide sequences of LmDEFs exhibit the major features of insect defensins including a signal sequence or the pre-prodefensins, followed by a propeptide sequence at the *N*-terminus (immature peptide; rich in aspartic and glutamic residues in both LmDEF 1 and 3), and the mature defensin peptide at the C-terminus. A dibasic cleavage site (-KR↓), which is essential in the post-translational proteolytic processing of mature peptide precursor, was recognized for LmDEF5. The conserved dibasic cleavage sites (-KR- and -RR-) have been found in many defensin sequences identified from different insect orders, including Anoplura, Heteroptera, Diptera, and Coleoptera [3, 4]. The consensus motifs of LmDEFs is consistent with the spacing pattern of insect defensins (C-X_5–16_-C-X_3_-C-X_9–11_-C-X_4–7_-C-X_1_-C) [57]. The strategy used for transcriptome analysis and mining was very effective, with the identification of four new defensins. It is noteworthy that Zhang *et al*. [36] identified only one unigene corresponding to a defensin in the overall transcriptome library (fat body and hemocytes) of *M*. acridum-infected *L*. *migratoria*.

Evolutionary relationships with other insect defensin inferred from the NJ phylogeny showed that LmDEF 1 and 3 are more closely related to Hymenoptera defensins than to other exopterygote defensins, although locusts are phylogenetically distant from bees and wasps. A similar phenomenon was reported for c-type lysozymes of both *Shistocerca gregaria* and *L*. *migratoria* [58]. However, LmDEF5 is convergently related to the exoptergotes. We suggest that hymenopteran defensins and LmDEF 1 and 3 possibly perform comparable *in vivo* functions due to the close relatedness in both sequence similarities and phylogeny analysis. Nevertheless, the inadequate availability of molecular data on the evolution of invertebrate defensins cannot provide an upfront explanation of the evolutionary history of these defensins and AMPs in the broad sense [59].

Remarkably, we initially located the *LmDEFs* in the labial and maxillary palpi transcriptome of *Locusta*. Such unique presence of AMPs such as defensins in epidermal cells of these olfactory organs was reported before in *Drosophila*. Local expression of a drosomycin-GFP reporter gene in the epidermal cells of the maxillary palps in the absence of experimental immune challenge was pronounced [60]. Also, one of the divergently and differentially expressed genes in heads of *D*. *sechellia* was defensin induced by immune challenges [61].

### Structure

The topologies of locust defensins could explain their putative toxic effect, and this is supported by their induced expression upon infection. Defensins of the CSαβ-type share an amphipathic secondary structure with a polar cationic site, hence, the terms “cell-penetrating peptides” cover their inhibitory activities on the microbial levels through a membrane disruptive mode of action [16]. Taking into account both *Metarhizium* and the microsporidian (a fungi-related group) *N*. *locustae*, the antifungal action of insect defensins is based on interaction with particular sphingolipids in membranes and cell walls of susceptible fungi. Sphingolipids are an important structural component of eukaryotic membranes and are also recognized as secondary messenger molecules regulating the equilibrium between cell death and cell growth processes [62]. Both ceramides and glycosphingolipids are present ubiquitously as essential membrane components in almost all eukaryotic cells and mitochondria [63]. The interaction with fungal cell wall glucosylceramide also has been reported for the antifungal CSαβ-type defensin heliomicin from *Heliothis virescens* [64]. Interestingly, LmDEF sequence searches versus existing PDB entries, using PDB files, showed significant homologues to known defensins with broad-spectrum activities. The 3D structure of LmDEF1 showed 50% identity to that of defensin A from *Phormia terranovae* larvae, an antibacterial that targets the bacterial cytoplasmic membrane [8], 47.5% identity with sapecin, a defensin with both antibacterial membrane permeabilization activity and prominent fungicidal actions [65, 66], and 48.8% identity with coprisin, an antibacterial and antifungal peptide with pro-apoptotic effects [14]. The LmDEF3 3D structure model showed 60, 59, and 57.5% identities to the previous defensins, respectively. Meanwhile, LmDEF5 showed 50% identity to bmbktx1, a cationic and Cys-rich potassium channel blocker from the scorpion *Buthus martensi* that forms a typical CSαβ scaffold adopted by most insect defensins [67]. In the compared 3D models, efforts were made to understand how the structural motifs would support protein functionality. Their alpha-helices are amphipathic, which bury their hydrophobic residues in the hydrophobic part of the bilayer while the polar/charged residues should stay at the level of the lipid polar headgroups.

Also, a comparison between LmDEFs and the antiparasitic *Ph*. *duboscqi* defensin (PhdDEF) [11] at the sequence level was conducted to explain a possible antiparasitic potential (Fig 3). PhdDEF is a broad-spectrum antimicrobial and antiparasitic defensin. It was induced by bacterial and parasitic hemolymph and gut infections. Expression peaks at 24 hours post infection by the bacterium *Pectobacterium carotovorum*, and at day four post infection by the parasite *Leishmania major*. It is synthesized by the fat body and eventually secreted into the hemolymph and has antiparasitic activity against promastigote forms of *L*. *major*, and antibacterial activity against Gram-positive bacterium *S*. *aureus*. PhdDEF has antifungal activity against the yeasts *Candida albicans* and *Saccharomyces cerevisiae*, but not *C*. *glabrata*, and has antifungal activity against filamentous fungi *Aspergillus fumigatus*, *Fusarium culmorum*, *F*. *oxysporum*, *Neurospora crassa*, *Trichoderma viride* and *Trichophyton mentagrophytes*, but not *Beauveria bassiana* (*see*, UniProtKB/Swiss-Prot entry P83404.3). In the future, the *in vitro* and *in vivo* actions of LmDEFs on microsporidia and their antimicrobial potential overall need to be clarified.

### Dynamics of LmDEFs expression in locust tissues after immune-challenge

The locust immune response to parasitism of *N*. *locustae* and mycosis with *Metarhizium* was analyzed in a triplicate-repeated independent experiment with conventional non-quantitative RT-PCR. Topical application of *Metarhizium anisopliae* var *acridum* to nymphs resulted in changes in the mRNA expression of *LmDEFs*. *M*. *anisopliae* var *acridum* colonize locust hosts from day two post application [68]. In our experiments, the fungal infection appeared to stimulate transcription and biosynthesis of defensins over the first few days of mycosis though the level declined subsequently. The differential expression showed an initial increase in gene expression that may be primarily due to a larger number of conidial and hyphal bodies within the locust host. After day five, consistent declines in transcript levels were observed in mycosed hoppers. Levels of the tested *LmDEFs* were significantly lower in control locusts than in infected ones.

A correlation was found between gene expression changes in experiments showing that the salivary glands and fat body transcriptional responses to infection were reproducible. Defensin transcripts were mainly upregulated in progression of infection, hence, the amplification regarding gene expression changes in this latent transcriptional profile might reflect the ability of diseased locusts to recover from this disruption or in broad sense are able to circumvent the stress caused by parasite metabolites, taking into consideration that the effect of locust ageing on gene expression profiles was corrected (data not shown). Moreover, to ascertain the induced nature of *LmDEFs* in response to *Nosema* infection, the gene expression profiles of *LmDEF* 1, 3, and 5 were compared between control locusts and *Nosema*-inoculated nymphs at 3, 5, 10 and 15 days post-inoculation. The results showed that the mRNA levels of *LmDEFs* were mainly upregulated in *N*. *locustae*-infected nymphs. In contrast to our results, a defensin unigene of 246 nt has been identified in the overall transcriptome library of *M*. *acridum*-infected *L*. *migratoria*, but no differential expression of it was observed in either immune-challenged fat body or hemocytes [36].

We investigated whether *N*. *locustae* induces the biosynthesis of locust defensins. qRT-PCR analysis of the transcriptional profile of *LmDEFs* in both fat body and salivary glands under nosemosis suggested that *Nosema* development, supported by localizing the *Nosema β-actin* at day 10 post-infection, may have a growing impact on the immune gene expression changes evidenced by augmented transcript levels at day 15 compared to day 5. At the time of establishing infection (merogony), *Nosema* is in a meront phase with no outer membrane, the later forming during the sporogonic phase of development [69, 70]. The major glycoconjugates in the polar sac-anchoring disc complex or “polar cap” and the spore wall of *Nosema* are the *O*-linked glycans (mainly manno-oligosaccharides), resembling those reported in some fungi such as *C*. *albicans*, which play a major role in host-parasite interactions and pathogenic determinants [71]. These glycans act as elicitors of the innate immune system [72], and consequently, activate the transcription of many immune genes, including the NFkappaB/Rel regulated defensins [73]. In *Anopheles gambiae*, the DEF1 promoter activity is affected by other proteins in the NFkappaB/Rel pathway and the Imd pathway [74, 75]. It is worth mentioning that, the Toll pathway, which is thought to mainly respond to fungi and to some extent to Gram-positive bacteria, is activated within hours of infection, and the transcription of target genes, such as the antifungal drosomycin, persists for days [76, 77]. Hence, the slowly-activated Toll pathway response is likely more effective against slow-replicating pathogens, such as fungi and protozoa.

Finally, here we show that the transcription of defensins was increased in infected locusts, and hence, *Locusta* defensins might display antimycotic and protozoacidal activities against forms of *M*. *anisopliae* and *N*. *locustae*. The differentially expressed transcripts and reduced locust susceptibility to both *Nosema locustae* and *M*. *anisopliae* were confirmed at the transcriptional level. *Locusta* defensins are innate immune molecules that play an important role in reducing infection. The discovery of new AMPs induced by protozoan pathogens in their insect vectors raises the possibility that they play an important role during parasitic infections [11]. The basic knowledge derived from these analyses may direct us not only to a more rational development of insect pathogens for biological control but also to investigate the possibility of genetically manipulating the target insect’s immune system itself.

## Conclusions

In this work, four defensin encoding transcripts from the migratory locust *L*. *migratoria* were identified and characterized. An isolation strategy based on homologous sequences encoding mature defensins from other insect species was used to isolate these sequences, three of which were shown to be novel for exoptergote defensins of the CSαβ structural group. Expression analyses showed that three peptides have differential expression patterns in the tested tissues in a time-series approach. These patterns suggest a role in protecting the locust host against pathogenic attack, or roles in unknown physiological processes in these tissues. Furthermore, the evolutionary divergences and convergences in structural motifs observed for these peptides bring future attention to study structure-activity correlations and determinants in these peptides. Finally, the results described here represent a substantial addition to our understanding of the immune repertoire of *L*. *migratoria*.

## Supporting Information

S1 Fig Preliminary screening for peptide(s) exhibiting antibacterial activity. (DOCX)

S2 Fig cDNAs and predicted amino acid sequences of LmDEFs. (DOCX)

S3 Fig qRT-PCR additional data. (DOCX)

S4 Fig Comparative relative expression levels, with the statistical annotations, of *LmDEFs* transcripts in the fat body and salivary glands of *Nosema*-infected nymphs by qRT-PCR. (DOCX)

S1 Table Defensins used in homology searches. (DOCX)

S2 Table List of primers used to identify and validate the defensin genes in this study. (DOCX)

S3 Table Secondary structure elements of LmDEFs obtained from ProFunc server at EMBL-EBI. (DOCX)

S4 Table Statistics for selected PDB entries that matched the sequences of PDBs of LmDEFs 1, 3, and 5 against all protein sequences in the PDB. (DOCX)

S5 Table LmDEFs sequence searches *vs* existing PDB entries, using the EMBL-EBI ProFunc server. (DOCX)
