# Supplementary material for: A Family of CSαβ Defensins and Defensin-Like Peptides from the Migratory Locust, Locusta migratoria, and Their Expression Dynamics during Mycosis and Nosemosis
Source: PLoS One. 2016 Aug 24;11(8):e0161585. doi: 10.1371/journal.pone.0161585 (PMC4996505; doi:10.1371/journal.pone.0161585)
Supplement: S1 Fig — (DOCX) [file pone.0161585.s001.docx]

a **
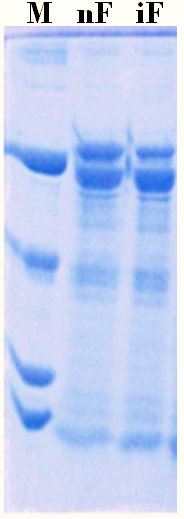
** b
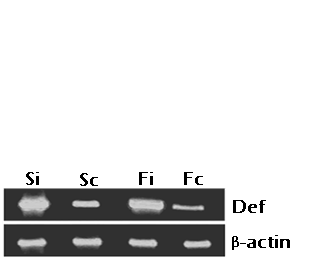
 c

**S1 Fig.** **Preliminary screening for peptide(s) exhibiting antibacterial activity**. a, Levels of antibacterial activity recorded from fat body preparations of 5^th^ instar *L*. *migratoria*. Activity was assessed using a zone of inhibition test against both *E*. *coli* and *S*. *aureus*. Each value represents the mean ± SD of the readings from three nymphs (n=3), means with different letters are significantly different at *P*<0.05. b, 12% SDS-PAGE of native (nF) and *Serratia*-induced (iF) fat body preparation from 5^th^ instar *L*. *migratoria*. M, protein marker (175-7 kDa; New England Biolabs, USA, NEB #P7703). c, Relative expression of partially-isolated defensin gene in salivary gland (S) and fat body cells (F) treated with bacteria or induced (i) and naïve controls (c), normalized to *β-actin*.
