# Supplementary material for: A Family of CSαβ Defensins and Defensin-Like Peptides from the Migratory Locust, Locusta migratoria, and Their Expression Dynamics during Mycosis and Nosemosis
Source: PLoS One. 2016 Aug 24;11(8):e0161585. doi: 10.1371/journal.pone.0161585 (PMC4996505; doi:10.1371/journal.pone.0161585)
Supplement: S2 Fig — (DOCX) [file pone.0161585.s002.docx]

>*LmDEF1*, c269909.graph_c0, ORF 237bp

GTTTGCTACACTGCTGAAGCAGACGCTCCCTTTGAGACGACACCACCATGAAGACGTCGACGGTCGCCTTTCTTTTCGCGCTGCTCGCTGTTGCCAGTATTTCCTCTTCAGCGCCAGTGGAGGAGGAGCAGCATGAGCACGGCGACCGCCACGTGCGCTTCACGTGTGACGCGCTGAGCGCCTTCGGCGTGGCGGACACTGCCTGTGCTGTCCGCTGCGTGGCGATGAACAGAGGATATGCGGGAGGACACTGCGACCAGGGCGTCTGCCACTGCCGCAAGTAGGGGCAGCCAGCACAGCGAGGAACCTGCATGCTGTGCTTCATCAGCACAATGCTGGACACTGTGCTGCCATATTGTGACACACTATACATTTCTGTAATATTTTTATATTTTTATAGTGAACTGACATG**AATAAA**GAAAAAATACAAGGGAAAAAAAAAAAAAAAAC

ORF protein translation (78 aa):

48 atgaagacgtcgacggtcgcctttcttttcgcgctgctcgctgtt

**M K T S T V A F L F A L L A V**

93 gccagtatttcctcttcagcgccagtggaggaggagcagcatgag

**A S I S S S**↓ A P V E E E Q H E

138 cacggcgaccgccacgtgcgcttcacgtgtgacgcgctgagcgcc

H G D R H V R F T C D A L S A

183 ttcggcgtggcggacactgcctgtgctgtccgctgcgtggcgatg

F G V A D T A C A V R C V A M

228 aacagaggatatgcgggaggacactgcgaccagggcgtctgccac

N R G Y A G G H C D Q G V C H

273 tgccgcaagtag 284

C R K *

>*LmDEF3*, c277049.graph_c0, ORF 237bp

GTGGCTGAGCGGGCGTTTCCACAAACCAGCAGCTGCCGTTTGCTCTGTGGAAACGCCCGCTCAGCCACGATGAAACTCTCCGTCGCAGCGGTCCTTCTAGCCCTTGTCGTCTTCGCGTCTGTCTCCTCTGCTGCACCAGCAGCGGGAGGAGAGGAGGAGCACGGCGACCGACACGTGCGAGTCACGTGCGACCTGCTGAGCGCGTTCGGCGTGGAGCACTCTGCGTGCGCTGCGCACTGCATCGCCATGAACAAGGGCTACAAGGGAGGACACTGCGACGACGGCGTCTGCCACTGCCGCAAGTAGCAAGTGGCCATCTGAGGTCGCAGCCGTCTCCTCGCTGCCGACACTACATTCTTTCACTCACTGTACACAACTGTTGTCAATAAAACTTGTTTCTTGCCTGCTGATGTGCAAAAAAA

ORF protein translation (78 aa):

70 atgaaactctccgtcgcagcggtccttctagcccttgtcgtcttc

**M K L S V A A V L L A L V V F**

115 gcgtctgtctcctctgctgcaccagcagcgggaggagaggaggag

**A S V S S A**↓ A P A A G G E E E

160 cacggcgaccgacacgtgcgagtcacgtgcgacctgctgagcgcg

H G D R H V R V T C D L L S A

205 ttcggcgtggagcactctgcgtgcgctgcgcactgcatcgccatg

F G V E H S A C A A H C I A M

250 aacaagggctacaagggaggacactgcgacgacggcgtctgccac

N K G Y K G G H C D D G V C H

295 tgccgcaagtag 306

C R K *

*>LmDEF4*, c274387.graph_c0, ORF 210bp

TGCAAGAAACCTCGTGCGAGCAGATATCACCATGAAGAATTCCACTGTGTTCTTTTTAGTCGGTCTCCTTACCACTGCTGGCATCGCCTTTTGTTCTGCAGCACCGGCGCAGAGTGTGCAGGACGACCGCCAGGCGCACCTCACGTGTGACTCGCTGAGCGCCTTGGGCGTGCCGTGCGCCGCCGTCCGCTGCGTCAAGGGCGCCTACTGCCAGCACGGCGTCTGCCACTGCCGCGTGTAGGCGTATCCCAGCACGGCGCAGCAGCTGGGAAACACCACTGCTCACTGACATGTGCTCTTCACAAATAACGTGCCCCACACTGCAGTGCATGCCTGTTGTTTTTGTGCAACCAAGAGAAATTTATATACAAGCTGAAGAACTTAACCATTCTATGCATGACCTATATTTTT

ORF protein translation (69 aa):

32 atgaagaattccactgtgttctttttagtcggtctccttaccact

**M K N S T V F F L V G L L T T**

77 gctggcatcgccttttgttctgcagcaccggcgcagagtgtgcag

**A G I A F C S A**↓ A P A Q S V Q

122 gacgaccgccaggcgcacctcacgtgtgactcgctgagcgccttg

D D R Q A H L T C D S L S A L

167 ggcgtgccgtgcgccgccgtccgctgcgtcaagggcgcctactgc

G V P C A A V R C V K G A Y C

212 cagcacggcgtctgccactgccgcgtgtag 241

*>LmDEF5*, c280465.graph_c0, ORF 204bp

CGGCAGTGGAGAGATGGCGGCGGTCGCGGTTCTGCTGCTGGCACTGGCACTGCTCGCATCCGCAGCTGACGCAGGCACGGTGAGAATGAAGCGCGGGACGTGCGACAACAGCATCGACGAGATGAACAGGCTGGCATGCAACGCATACTGCGTGGCGCAGCTGAACGGCTACTCCGGCGGCAAGTGCCTGCTGGGCACCTGCGTCTGCTACAAGTAGGCCGGCACCCTGCGAGGCCACACCATGGGAACCGCACTTCACGTAGTGAAAAATTCTGCCAGCACTACACTCAGTAATTATAGTGTTGTAGTATTTGCA**AATAAA**TGATAAAAATAAAAAGTCTATCTGTAACAAGTAGGCCGCACTAGTTGTGGCAGGCAATTCACGAGGTCACACCATGGGAACGGCA

ORF protein translation (67 aa):

14 atggcggcggtcgcggttctgctgctggcactggcactgctcgca

**M A A V A V L L L A L A L L A**

59 tccgcagctgacgcaggcacggtgagaatgaagcgcgggacgtgc

**S A A D A**↓ G T V R M K R G T C

104 gacaacagcatcgacgagatgaacaggctggcatgcaacgcatac

D N S I D E M N R L A C N A Y

149 tgcgtggcgcagctgaacggctactccggcggcaagtgcctgctg

C V A Q L N G Y S G G K C L L

194 ggcacctgcgtctgctacaagtag 217

G T C V C Y K *

**S2 Fig. cDNAs and predicted amino acid sequences of LmDEFs.** cDNA sequence (upper) and predicted amino acid sequence (one letter code, below) of LmDEFs. Numerical designation of the starting position of ORF nucleotide sequence is shown on the left and right. Assigned initial and terminal codons are marked with teal and pink, respectively. The termination codon is marked with an asterisk (lower panel). Sequences corresponding to the 5’ and 3’ UTR regions are underlined and double-underlined, respectively. The bold face amino acid sequence indicates the predicted signal peptide and its cleavage site is indicated by a red arrow. The putative polyadenylation signal AATAAA is bold-shadowed and underlined. The complete coding sequences were deposited in GenBank and designated as *LmDEF1* [Genebank: [KU516092.1](http://www.ncbi.nlm.nih.gov/nucleotide/1003776869?report=genbank&log$=nucltop&blast_rank=2&RID=JD2FDXUU01R)], *LmDEF3* [Genebank: [KU516094.1](http://www.ncbi.nlm.nih.gov/nucleotide/1003777038?report=genbank&log$=nucltop&blast_rank=1&RID=JD2FR6NS01R)], *LmDEF4* [Genebank: [KU516095.1](http://www.ncbi.nlm.nih.gov/nucleotide/1003777105?report=genbank&log$=nucltop&blast_rank=1&RID=JD2FDXUU01R)], and *LmDEF5* [Genebank: [KU516096.1](http://www.ncbi.nlm.nih.gov/nucleotide/1003777165?report=genbank&log$=nucltop&blast_rank=1&RID=JD2EYFXC01R)].
