# Supplementary material for: A Family of CSαβ Defensins and Defensin-Like Peptides from the Migratory Locust, Locusta migratoria, and Their Expression Dynamics during Mycosis and Nosemosis
Source: PLoS One. 2016 Aug 24;11(8):e0161585. doi: 10.1371/journal.pone.0161585 (PMC4996505; doi:10.1371/journal.pone.0161585)
Supplement: S3 Fig — (DOCX) [file pone.0161585.s003.docx]

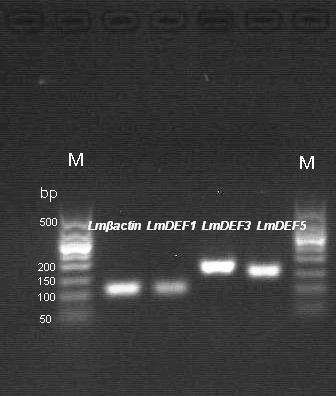


a


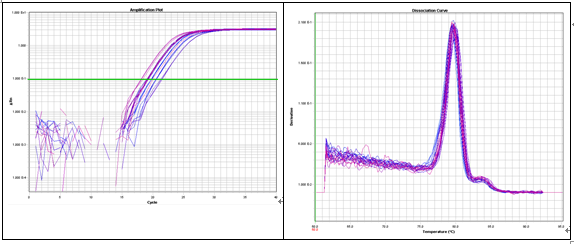


*Lmβactin*


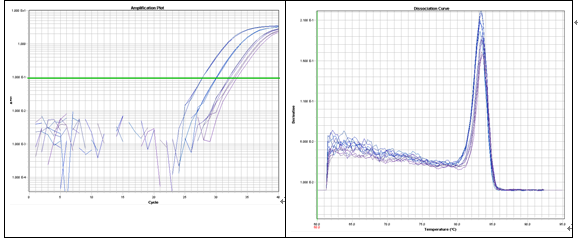


*LmDEF1*


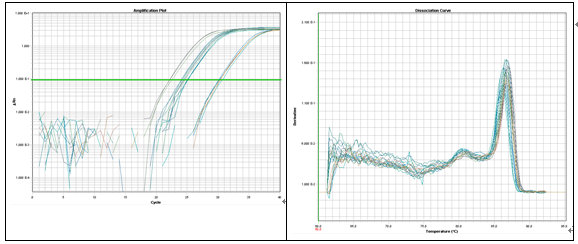


*LmDEF3*


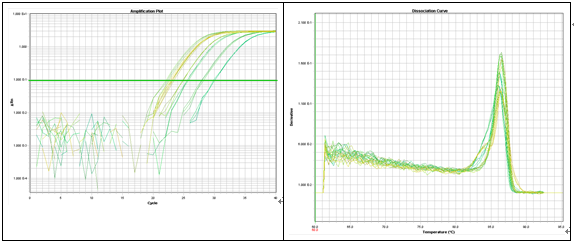


*LmDEF5*

b


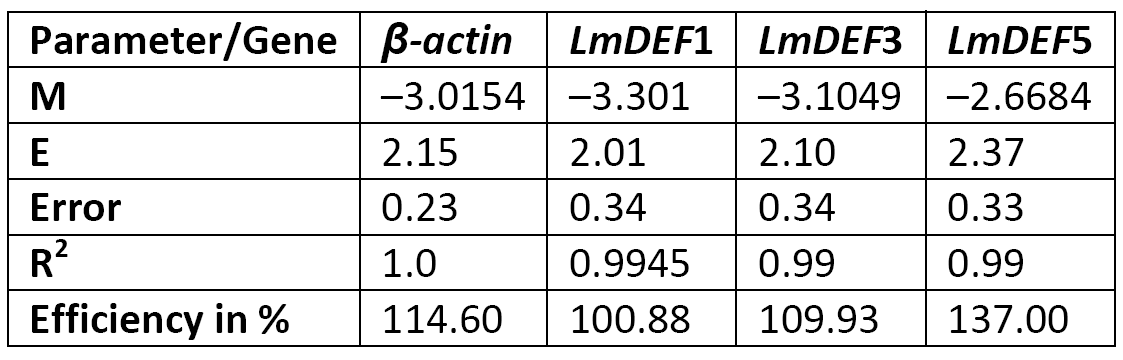


c

**S3 Fig. qRT-PCR additional data.** a, Gel analysis of amplification products. M (left&right), 50bp DNA Ladder. b, The amplification curve (left) in qRT-PCR and product melt curve (right) of *Lmβactin*, *LmDEF1*, *LmDEF3*, and *LmDEF5*. c, qPCR quality control data for each reaction. M, the expected average quantification cycle (Cq) between each 10-fold standard curve dilution (optimal M= –3.3); E, efficiency of the RT-qPCR reaction (optimal E= 1.00), and R2, correlation coefficient (optimal R2 is equal to 1).
