# Supplementary material for: A Family of CSαβ Defensins and Defensin-Like Peptides from the Migratory Locust, Locusta migratoria, and Their Expression Dynamics during Mycosis and Nosemosis
Source: PLoS One. 2016 Aug 24;11(8):e0161585. doi: 10.1371/journal.pone.0161585 (PMC4996505; doi:10.1371/journal.pone.0161585)
Supplement: S4 Fig — (DOCX) [file pone.0161585.s004.docx]

*LmDEF1*

*LmDEF1*

| 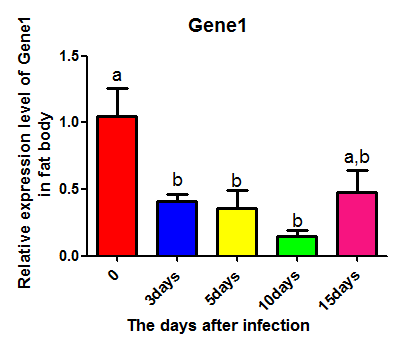 | 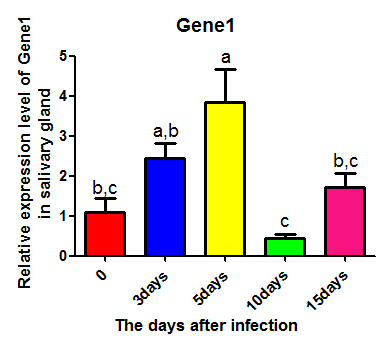 |
| --- | --- |
| 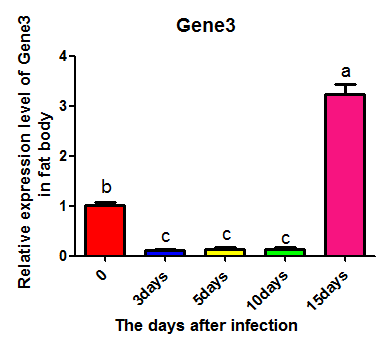  *LmDEF3* | 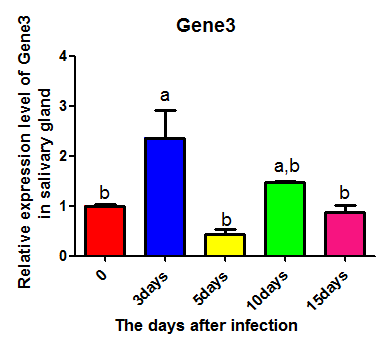  *LmDEF3* |
| 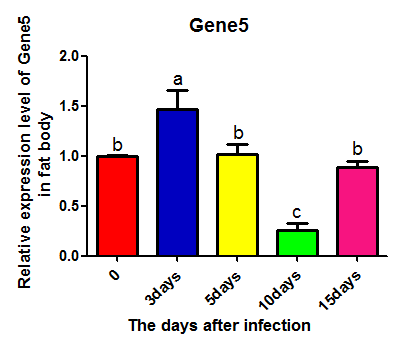  *LmDEF5* | 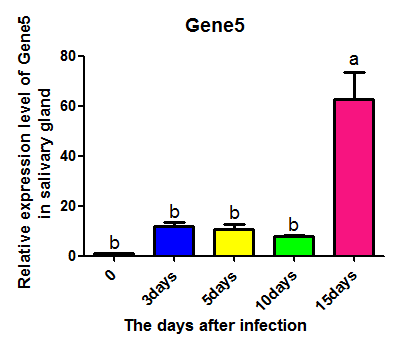  *LmDEF5* |

**Fat body Salivary glands**

**S4 Fig.** **Comparative relative expression levels, with statistical annotations, of *LmDEF* transcripts in fat body and salivary glands of *Nosema*-infected nymphs by qRT-PCR.** The expression levels of non-infected tissues were used as controls. Values are means ± SE, n=3 for each group. Different letters above columns indicate statistically significant differences at *p*<0.05 level (Tukey's HSD post-hoc tests).
