# Supplementary material for: A Family of CSαβ Defensins and Defensin-Like Peptides from the Migratory Locust, Locusta migratoria, and Their Expression Dynamics during Mycosis and Nosemosis
Source: PLoS One. 2016 Aug 24;11(8):e0161585. doi: 10.1371/journal.pone.0161585 (PMC4996505; doi:10.1371/journal.pone.0161585)
Supplement: S1 Table — (DOCX) [file pone.0161585.s005.docx]

**Table S1.** Defensins used in homology searches

| **Species** | **Order** | **Accession number** | **Reference** |
| --- | --- | --- | --- |
| *Acalolepta luxuriosa* | Coleoptera | [AAK35160.1](http://www.ncbi.nlm.nih.gov/protein/13625795) | Ueda et al. (2005) Appl. Entomol. Zool. (Jpn.) 40 (2), 335-345 |
| *Acromyrmex echinatior* | Hymenoptera | [XP_011055302.1](http://www.ncbi.nlm.nih.gov/protein/746849699) | NCBI eukaryotic genome annotation pipeline |
| *Aedes aegypti* | Diptera | [AAD40112.1](http://www.ncbi.nlm.nih.gov/protein/5107399) | Lowenberger et al. (1999) Insect Mol. Biol. 8 (1), 107-118 |
| *Aeshna cyanea* | Odonata | [P80154](http://www.uniprot.org/uniprot/P80154) | Bulet et al. (1992) Eur. J. Biochem. 209 (3), 977-984 |
| *Anomala cuprea* | Coleoptera | [BAD77967.1](http://www.ncbi.nlm.nih.gov/protein/56565273) | Yamauchi (2004) - Direct submission - |
| ***Anopheles gambiae*** | **Diptera** | [**ABB00983.1**](http://www.ncbi.nlm.nih.gov/protein/77702038) | **Simard et al. (2005) - Direct submission -** |
| *Apis cerana cerana* | Hymenoptera | [ACH96408.1](http://www.ncbi.nlm.nih.gov/protein/202078692) | Xu et al. (2009) PLoS ONE 4 (1), E4239 |
| *Apis mellifera* | Hymenoptera | [AGM19140.1](http://www.ncbi.nlm.nih.gov/protein/506614548) | Harpur and Zayed (2013) Mol. Biol. Evol. 30 (7), 1665-1674 |
| ***Apis mellifera*** | **Hymenoptera** | [**ACT66903.1**](http://www.ncbi.nlm.nih.gov/protein/254548155) | **Viljakainen et al. (2009) Mol. Biol. Evol. 26 (8), 1791-1801** |
| *Bombus ignitus* | Hymenoptera | [AAQ90412.1](http://www.ncbi.nlm.nih.gov/protein/37256234) | Byeon et al. (2003) - Direct submission - |
| *Bombyx mori* | Lepidoptera | [BAG48202.1](http://www.ncbi.nlm.nih.gov/protein/189547731) | Wen et al. (2009) Mol. Biol. Rep. 36 (4), 711-716 |
| *Camponotus floridanus* | Hymenoptera | [EFN62355.1](http://www.ncbi.nlm.nih.gov/protein/307169846) | Bonasio et al. (2010) Science 329 (5995), 1068-1071 |
| *Copris tripartitus* | Coleoptera | [ABP97087.1](http://www.ncbi.nlm.nih.gov/protein/145864817) | Hwang et al. (2006) - Direct submission - |
| ***Cotesia vestalis*** | **Hymenoptera** | [**AGE89781.1**](http://www.ncbi.nlm.nih.gov/protein/449138902) | **Wang et al. (2013) J. Insect Physiol. 59 (11), 1095-1103** |
| *Culex quinquefasciatus* | Diptera | [AFI81525.1](http://www.ncbi.nlm.nih.gov/protein/385845654) | Wang and Zhang (2012) - Direct submission - |
| *Danaus plexippus* | Lepidoptera | [EHJ63539.1](http://www.ncbi.nlm.nih.gov/protein/357602863) | Zhan et al. (2011) Cell 147 (5), 1171-1185 |
| *Drosophila melanogaster* | Diptera | [AAO72500.1](http://www.ncbi.nlm.nih.gov/protein/29367272?report=genbank&log$=prottop&blast_rank=1&RID=8DGDJCJA01R) | Lazzaro and Clark (2003) Mol. Biol. Evol. 20 (6), 914-923 |
| *Drosophila melanogaster* (X) | Diptera | [1MYN_A](http://www.ncbi.nlm.nih.gov/protein/159162723?report=genbank&log$=prottop&blast_rank=16&RID=8DHVAAVJ01R) | Landon et al. (1997) Protein Sci. 6 (9), 1878-1884 |
| *Galleria mellonella* | Lepidoptera | [P85213.2](http://www.ncbi.nlm.nih.gov/protein/254763283?report=genbank&log$=prottop&blast_rank=1&RID=8DJ2PKN701R) | Cytrynska et al. (2007) Peptides 28 (3), 533-546 |
| ***Graminella nigrifrons*** | **Orthoptera** | [**AIY24634.1**](http://www.ncbi.nlm.nih.gov/protein/723941665) | **Chen et al. (2013) - Direct submission -** |
| *Musca domestica* | Diptera | [ABM66377.1](http://www.ncbi.nlm.nih.gov/protein/122703768) | Xu et al. (2004) Am. J. Trop. Med. Hyg. 4, 513-517 |
| *Nilaparvata lugens - A* | Homoptera | [AGK40895.1](http://www.ncbi.nlm.nih.gov/protein/482875731) | Bao et al. (2013) BMC Genomics 14, 160 |
| *Nilaparvata lugens - B* | Homoptera | [AGK40896.1](http://www.ncbi.nlm.nih.gov/protein/482875733) | Bao et al. (2013) BMC Genomics 14, 160 |
| *Oryctes rhinoceros* | Coleoptera | [BAA36401.1](http://www.ncbi.nlm.nih.gov/protein/4115517) | Ishibashi et al. (1999) Eur. J. Biochem. 266 (2), 616-623 |
| ***Pediculus humanus corporis*** | **Phthiraptera** | [**XP_002432619.1**](http://www.ncbi.nlm.nih.gov/protein/242024407) | **Kirkness et al. (2007) - Direct submission -** |
| ***Pyrrhocoris apterus*** | **Hemiptera** | [**AGI17576.1**](http://www.ncbi.nlm.nih.gov/protein/471647537) | **Bajgar et al. (2013) Proc. Natl. Acad. Sci. U.S.A. 110 (11), 4416-4421** |
| *Reticulitermes virginicus* | Isoptera | [ADJ19003.1](http://www.ncbi.nlm.nih.gov/protein/299480768) | Bulmer et al. (2010) Insect Mol. Biol. 19 (5), 669-674 |
| *Rhodnius prolixus* | Hemiptera | [AAO74626.1](http://www.ncbi.nlm.nih.gov/protein/29335962) | Lopez et al. (2003) Insect Biochem. Mol. Biol. 33 (4), 439-447 |
| *Sitophilus zeamais* | Coleoptera | [ABZ80665.1](http://www.ncbi.nlm.nih.gov/protein/167444208) | Anselme et al. (2008) BMC Biol. 6, 43 |
| *Spodoptera exigua* | Lepidoptera | [AEW24427.1](http://www.ncbi.nlm.nih.gov/protein/363497927) | Hwang and Kim (2010) - Direct submission - |
| *Stomoxys calcitrans* | Diptera | [AAD56536.1](http://www.ncbi.nlm.nih.gov/protein/5924287) | Munks et al. (1999) - Direct submission - |
| *Thermobia domestica* | Thysanura | [CAM36306.1](http://www.ncbi.nlm.nih.gov/protein/133916472) | Altincicek and Vilcinskas (2007) Insect Biochem. Mol. Biol. 37 (7), 726-731 |
| *Triatoma brasiliensis* | Hemiptera | [AAV48636.1](http://www.ncbi.nlm.nih.gov/protein/55247597) | Araujo et al. (2006) Insect Biochem. Mol. Biol. 36 (7), 547-560 |
| *Triatoma infestans* | Hemiptera | [ABD61004.1](http://www.ncbi.nlm.nih.gov/protein/89112752) | Topal et al. (2006) - Direct submission - |
| *Trichoplusia ni* | Lepidoptera | [ABV68852.1](http://www.ncbi.nlm.nih.gov/protein/157704327) | Freitak et al. (2007) BMC Biol. 5, 56 |
