# Supplementary material for: A Family of CSαβ Defensins and Defensin-Like Peptides from the Migratory Locust, Locusta migratoria, and Their Expression Dynamics during Mycosis and Nosemosis
Source: PLoS One. 2016 Aug 24;11(8):e0161585. doi: 10.1371/journal.pone.0161585 (PMC4996505; doi:10.1371/journal.pone.0161585)
Supplement: S2 Table — (DOCX) [file pone.0161585.s006.docx]

**Table S2.** List of primers used to identify and validate the defensin genes in this study

| Primer name | Sequence 5'->3' | Annealing temperature (°C) |
| --- | --- | --- |
| LmPartialDEF-F | GCAGGCACGGTGAGAATGAA | 54.9 |
| LmPartialDEF-R | TCACTACGTGAAGTGCGGT | 52.5 |
| Non-quantitative RT-PCR  Lmig-ORF-1-F | TGCTCGCTGTTGCCAGTATTTC | 54.8 |
| Lmig-ORF-1-R | CCCGCATATCCTCTGTTCATCG | 54.5 |
| Lmig-ORF-3-F | CCTTGTCGTCTTCGCGTCTGTC | 57.4 |
| Lmig-ORF-3-R | CGCAGTGTCCTCCCTTGTAGC | 57.5 |
| Lmig-ORF-4-F | GCTGGCATCGCCTTTTGTTC | 54.8 |
| Lmig-ORF-4-R | CTACACGCGGCAGTGGCAGA | 58.9 |
| Lmig-ORF-5-F | CAGGCACGGTGAGAATGAAGC | 55.8 |
| Lmig-ORF-5-R | GCACTTGCCGCCGGAGTAG | 58.9 |
| Lmig-β-actin-F | CGAAGCACAGTCAAAGAGAGGTA | 53.1 |
| Lmig-β-actin-R | GCTTCAGTCAAGAGAACAGGATG | 52.1 |
| N.LocustaeActin-F | GGCATTCCCAAGCACAAAGG | 54.2 |
| N.LocustaeActin-R | ACAGAACAGCCTGAATCGCA | 53.3 |
| MetarhiziumActin-F | ATGTGCAAGGCCGGTTTCGC | 58.9 |
| MetarhiziumActin-R | TACGAGTCCTTCTGGCCCAT | 54.7 |
| qRT-PCR  LmDEF1-F | CTGAAGCAGACGCTCCCTTT | 60 |
| LmDEF1-R | CACTGGCGCTGAAGAGGAAA |  |
| LmDEF3-F | TCGCCATGAACAAGGGCTAC |  |
| LmDEF3-R | CACATCAGCAGGCAAGAAACA |  |
| LmDEF5-F | GCAGGCACGGTGAGAATGAA |  |
| LmDEF5-R | GGCCTACTTGTAGCAGACGC |  |
| Lmβ-actin-F | AACCCAAAGGCAAACAGGGA |  |
| Lmβ-actin-R | TCCTACCGGACGCATACAGA |  |
