# Supplementary material for: A Family of CSαβ Defensins and Defensin-Like Peptides from the Migratory Locust, Locusta migratoria, and Their Expression Dynamics during Mycosis and Nosemosis
Source: PLoS One. 2016 Aug 24;11(8):e0161585. doi: 10.1371/journal.pone.0161585 (PMC4996505; doi:10.1371/journal.pone.0161585)
Supplement: S3 Table — (DOCX) [file pone.0161585.s007.docx]

**Table. S3.** Secondary structure elements of LmDEFs obtained from ProFunc server at EMBL-EBI

|  | **β sheet** | **β hairpin** | **β bulge** | **β strands** | **α helix** | **β turns** | **γ turns** | **Disulphides** |
| --- | --- | --- | --- | --- | --- | --- | --- | --- |
| **LmDEF1** | 1 | 1 | 1 | 2 | 1 | 6 | 1 | 3 |
| **LmDEF3** | 1 | 1 | - | 2 | 1 | 6 | - | 3 |
| **LmDEF4** | 1 | 1 | - | 2 | 1 | 2 | - | - |
| **LmDEF5** | 1 | 1 | 1 | 2 | 1 | 6 | - | 3 |
