# Supplementary material for: A Family of CSαβ Defensins and Defensin-Like Peptides from the Migratory Locust, Locusta migratoria, and Their Expression Dynamics during Mycosis and Nosemosis
Source: PLoS One. 2016 Aug 24;11(8):e0161585. doi: 10.1371/journal.pone.0161585 (PMC4996505; doi:10.1371/journal.pone.0161585)
Supplement: S4 Table — (DOCX) [file pone.0161585.s008.docx]

**Table S4.** Statistics for selected PDB entries that matched the sequences of PDBs of LmDEFs 1, 3, and 5 against all protein sequences in the PDB

LmDEF1

|  | **Smith- Waterman score** |  | **%-tage identity** |  | **a.a. overlap** |  | **Seq len** |  | ***z*-score** |  | ***E*- value** |  |  | **PDB code** |  | **Entry name** |  |  |  |
| --- | --- | --- | --- | --- | --- | --- | --- | --- | --- | --- | --- | --- | --- | --- | --- | --- | --- | --- | --- |
|  | 158 |  | 48.7% |  | 39 |  | 41 |  | 277.7 |  | 1.9e-08 |  |  | [2ny9:X](http://www.ebi.ac.uk/pdbsum/2ny9) |  | *Nmr structure of def-abb, a mutant of anopheles defensin def-aaa* |  |  |  |
|  | 141 |  | 50.0% |  | 40 |  | 40 |  | 249.5 |  | 7.1e-07 |  |  | [1ica:A](http://www.ebi.ac.uk/pdbsum/1ica) |  | *Refined three-dimensional structure of insect defensin a* |  |  |  |
|  | 141 |  | 50.0% |  | 40 |  | 40 |  | 249.5 |  | 7.1e-07 |  |  | [2ny8:X](http://www.ebi.ac.uk/pdbsum/2ny8) |  | *Nmr structure of antibacterial defensin def-aaa from the insect anopheles gambiae* |  |  |  |
|  | 133 |  | 47.5% |  | 40 |  | 40 |  | 236.1 |  | 3.9e-06 |  |  | [1l4v:A](http://www.ebi.ac.uk/pdbsum/1l4v) |  | *Solution structure of sapecin* |  |  |  |
|  | 133 |  | 48.8% |  | 43 |  | 43 |  | 235.4 |  | 4.3e-06 |  |  | [2ln4:A](http://www.ebi.ac.uk/pdbsum/2ln4) |  | *Insight into the antimicrobial activities based on the struc activity relationships of coprisin isolated from the dung b copris tripartitus* |  |  |  |
|  | 96 |  | 37.9% |  | 29 |  | 33 |  | 175.7 |  | 0.0091 |  |  | [2koz:A](http://www.ebi.ac.uk/pdbsum/2koz) |  | *Solution structure of nasonin-1* |  |  |  |

LmDEF3

|  |  | **Smith- Waterman score** |  |  | **%-tage identity** |  | **a.a. overlap** |  | **Seq len** |  | ***z*-score** |  | ***E*- value** |  |  | **PDB code** |  | **Entry name** |  |  |  |
| --- | --- | --- | --- | --- | --- | --- | --- | --- | --- | --- | --- | --- | --- | --- | --- | --- | --- | --- | --- | --- | --- |
|  |  | 190 |  |  | 61.5% |  | 39 |  | 41 |  | 325.0 |  | 4.4e-11 |  |  | [2ny9:X](http://www.ebi.ac.uk/pdbsum/2ny9) |  | *Nmr structure of def-abb, a mutant of anopheles defensin def-aaa* |  |  |  |
|  |  | 177 |  |  | 60.0% |  | 40 |  | 40 |  | 304.0 |  | 6.5e-10 |  |  | [1ica:A](http://www.ebi.ac.uk/pdbsum/1ica) |  | *Refined three-dimensional structure of insect defensin a* |  |  |  |
|  |  | 170 |  |  | 59.1% |  | 44 |  | 43 |  | 291.9 |  | 3.1e-09 |  |  | [2ln4:A](http://www.ebi.ac.uk/pdbsum/2ln4) |  | *Insight into the antimicrobial activities based on the struc activity relationships of coprisin isolated from the dung b copris tripartitus* |  |  |  |
|  |  | 162 |  |  | 60.0% |  | 40 |  | 40 |  | 279.5 |  | 1.5e-08 |  |  | [2ny8:X](http://www.ebi.ac.uk/pdbsum/2ny8) |  | *Nmr structure of antibacterial defensin def-aaa from the insect anopheles gambiae* |  |  |  |
|  |  | 91 |  |  | 46.2% |  | 26 |  | 33 |  | 164.8 |  | 0.037 |  |  | [2koz:A](http://www.ebi.ac.uk/pdbsum/2koz) |  | *Solution structure of nasonin-1* |  |  |  |
|  |  |  |  |  |  |  |  |  |  |  |  |  |  |  |  |  |  |  |  |  |  |

LmDEF5

|  | **Smith- Waterman score** |  | **%-tage identity** |  | **a.a. overlap** |  | **Seq len** |  | ***z*-score** |  | ***E*- value** |  |  | **PDB code** |  | **Entry name** |  |  |  |
| --- | --- | --- | --- | --- | --- | --- | --- | --- | --- | --- | --- | --- | --- | --- | --- | --- | --- | --- | --- |
|  | 97 |  | 50.0% |  | 26 |  | 31 |  | 172.1 |  | 0.015 |  |  | [1q2k:A](http://www.ebi.ac.uk/pdbsum/1q2k) |  | *Solution structure of bmbktx1 a new potassium channel blocker from the chinese scorpion buthus martensi karsch* |  |  |  |
|  | 93 |  | 46.2% |  | 39 |  | 40 |  | 164.0 |  | 0.041 |  |  | [1ica:A](http://www.ebi.ac.uk/pdbsum/1ica) |  | *Refined three-dimensional structure of insect defensin a* |  |  |  |
|  | 87 |  | 41.4% |  | 29 |  | 43 |  | 153.8 |  | 0.15 |  |  | [2ln4:A](http://www.ebi.ac.uk/pdbsum/2ln4) |  | *Insight into the antimicrobial activities based on the struc activity relationships of coprisin isolated from the dung b copris tripartitus* |  |  |  |
|  | 85 |  | 43.6% |  | 39 |  | 40 |  | 151.2 |  | 0.21 |  |  | [1l4v:A](http://www.ebi.ac.uk/pdbsum/1l4v) |  | *Solution structure of sapecin* |  |  |  |

Annotations given are for sequences above 30% sequence identity and overlap of at least 30 residues (or 3/4 of the length of the submitted sequence, if this is shorter), or E-value < 0.001. EBI-SAS protein database. **Or alternatively Table S5 for all defensins**.
