# Supplementary material for: A Family of CSαβ Defensins and Defensin-Like Peptides from the Migratory Locust, Locusta migratoria, and Their Expression Dynamics during Mycosis and Nosemosis
Source: PLoS One. 2016 Aug 24;11(8):e0161585. doi: 10.1371/journal.pone.0161585 (PMC4996505; doi:10.1371/journal.pone.0161585)
Supplement: S5 Table — (DOCX) [file pone.0161585.s009.docx]

**Table S5.** LmDEFs sequence searches *vs* existing PDB entries, using the EMBL-EBI ProFunc server

| **PDB code** | **E-value** | **%-tage id** | **Overlap** | **Entry ID** |
| --- | --- | --- | --- | --- |
| **LmDEF1**† | | | | |
| *1ica (A) | 1.80E-05 | 50 | 40 | Refined three-dimensional structure of insect defensin a |
| *2ny8 (X) | 1.80E-05 | 50 | 40 | Nmr structure of antibacterial defensin def-aaa from the insect *Anopheles* *gambiae* |
| *1l4v (A) | 8.10E-05 | 47.5 | 40 | Solution structure of sapecin |
| *2ln4 (A) | 8.70E-05 | 48.837 | 43 | Insight into the antimicrobial activities based on the structure activity relationships of coprisin isolated from the dung beetle *Copris* *tripartitus* |
| **LmDEF3**† | | | | |
| **1ica (A) | 5.00E-09 | 60 | 40 | Refined three-dimensional structure of insect defensin a |
| **2lld(A) | 2.00E-08 | 57.5 | 40 | The insect defensin lucifensin from *Lucilia* *sericata* |
| **2ln4 (A) | 2.10E-08 | 59.091 | 44 | Insight into the antimicrobial activities based on the structure activity relationships of coprisin isolated from the dung beetle *Copris* *tripartitus* |
| **1l4v (A) | 2.40E-08 | 57.5 | 40 | Solution structure of sapecin |
| **LmDEF4** | | | | |
| *2ny9 (X) | 0.00079 | 45.946 | 37 | Nmr structure of def-abb, a mutant of *Anopheles* defensin def-aaa |
| ***1ica (A) | 0.017 | 47.368 | 38 | Refined three-dimensional structure of insect defensin a |
| ***2ln4(A) | 0.063 | 44.186 | 43 | Insight into the antimicrobial activities based on the structure activity relationships of coprisin isolated from the dung b *Copris* *tripartitus* |
| **LmDEF5**† | | | | |
| ***3e8y (X) | 0.02 | 50 | 26 | X-ray structure of scorpion toxin bmbktx1 |
| ***1q2k (A) | 0.02 | 50 | 26 | Solution structure of bmbktx1 a new potassium channel blocker from the Chinese scorpion *Buthus* *martensi* |

Possible homologues in the PDB were shown above cut off 45% identity.

The hits are marked in astrikes according to their likelihood of being correct as follows: **certain matches, *probable matches, and ***possible matches.

†ProSiteProfile: PS51378 motif; invertebrate defensins family profile.

(A): The structure has been determined by nuclear magnetic resonance (NMR) spectroscopy; while, X referred to structures determined by X-ray crystallography.
